# Comparing the Effects of Rocaglates on Energy Metabolism and Immune Modulation on Cells of the Human Immune System

**DOI:** 10.3390/ijms24065872

**Published:** 2023-03-20

**Authors:** Susanne Schiffmann, Marina Henke, Michelle Seifert, Thomas Ulshöfer, Luise A. Roser, Francesca Magari, Hans-Guido Wendel, Arnold Grünweller, Michael J. Parnham

**Affiliations:** 1 Fraunhofer Institute for Translational Medicine and Pharmacology ITMP, Theodor-Stern-Kai 7, 60596 Frankfurt am Main, Germany; 2 Pharmazentrum Frankfurt/ZAFES, Institute of Clinical Pharmacology, Goethe-University Hospital Frankfurt am Main, Theodor-Stern-Kai 7, 60590 Frankfurt am Main, Germany; 3 Institute of Pharmaceutical Chemistry, Philipps-University Marburg, Marbacher Weg 6, 35032 Marburg, Germany; 4 Cancer Biology and Genetics Program, Memorial Sloan Kettering Cancer Center, New York, NY 10065, USA; 5 EpiEndo Pharmaceuticals ehf, Bjargargata 1, 102 Reykjavik, Iceland

**Keywords:** immune modulation, antiviral activity, eIF4A, rocaglates, translation initiation, energy metabolism, cytokines

## Abstract

A promising new approach to broad spectrum antiviral drugs is the inhibition of the eukaryotic translation initiation factor 4A (elF4A), a DEAD-box RNA helicase that effectively reduces the replication of several pathogenic virus types. Beside the antipathogenic effect, modulation of a host enzyme activity could also have an impact on the immune system. Therefore, we performed a comprehensive study on the influence of elF4A inhibition with natural and synthetic rocaglates on various immune cells. The effect of the rocaglates zotatifin, silvestrol and CR-31-B (−), as well as the nonactive enantiomer CR-31-B (+), on the expression of surface markers, release of cytokines, proliferation, inflammatory mediators and metabolic activity in primary human monocyte-derived macrophages (MdMs), monocyte-derived dendritic cells (MdDCs), T cells and B cells was assessed. The inhibition of elF4A reduced the inflammatory potential and energy metabolism of M1 MdMs, whereas in M2 MdMs, drug-specific and less target-specific effects were observed. Rocaglate treatment also reduced the inflammatory potential of activated MdDCs by altering cytokine release. In T cells, the inhibition of elF4A impaired their activation by reducing the proliferation rate, expression of CD25 and cytokine release. The inhibition of elF4A further reduced B-cell proliferation, plasma cell formation and the release of immune globulins. In conclusion, the inhibition of the elF4A RNA helicase with rocaglates suppressed the function of M1 MdMs, MdDCs, T cells and B cells. This suggests that rocaglates, while inhibiting viral replication, may also suppress bystander tissue injury by the host immune system. Thus, dosing of rocaglates would need to be adjusted to prevent excessive immune suppression without reducing their antiviral activity.

## 1. Introduction

The world has faced a number of severe virus outbreaks in recent years, such as the Zika virus outbreak in South America, the Ebola virus outbreak in West Africa, and the worldwide SARS-CoV-2 pandemic. However, treatment options for virus-mediated diseases are limited despite the new developments, such as nirmatrelvir that inhibits 3C-like protease of SARS-CoV-2. Moreover, the treatment options are often virus specific, have side effects and are only effective following early and timely treatment [1]. This points to the fact that well-tolerated and efficient broad-spectrum antiviral therapies are urgently needed [2].

Targeting the RNA helicase elF4A to unwind stable RNA secondary structures during translation initiation [3,4] seems to be a promising approach for the treatment of viral infections [5,6] since several highly pathogenic viruses rely on this host factor for the translation of their mRNAs. The group of specific elF4A helicase inhibitors comprises, among others, rocaglates like silvestrol, zotatifin and CR-31-B (−) [7,8,9].

Silvestrol is a complex natural compound that can be isolated from plants of the genus *Aglaia* [10]. Synthesis of silvestrol is time-consuming and expensive, and thus access to the drug is limited. However, synthetic rocaglates like zotatifin or CR-31-B (−) can be chemically synthesized. Rocaglates were initially described in the field of cancer research where they showed potent antitumor activity in vitro and in vivo [11,12,13]. Importantly, rocaglates are broad-spectrum antiviral compounds and efficiently inhibit the replication of subtypes of Corona-, Flavi-, Picorna-, Filo- and Togaviruses by blocking the synthesis of viral proteins [14,15,16,17,18,19,20]. Targeting of host factors has advantages, such as a decreased risk of escape mutations by the virus [21], but also presents difficulties compared to viral targets, such as possible pleiotropic side effects [22]. Rocaglates have been shown to be highly specific inhibitors of eIF4A by clamping the RNA substrate onto the surface of eIF4A [4,9,23]. Therefore, the risk of the potential side effects from rocaglate treatment should be low since other RNA helicases are not affected by this compound class.

Viruses infect the host, which fights back against the infection via an innate and adaptive immune system to restore health. However, sometimes viruses activate the immune system to an excessive degree and instead of combating the virus, the immune system attacks host tissues. In this context, it is important to determine whether an antiviral compound can also mediate an immune-modulatory effect. The antiviral response can support the immune system in combatting the virus, but it can also downregulate an overreacting immune response to prevent tissue from injury. Such modulation of the immune system can broaden the drug efficacy profile by boosting innate host defense mechanisms without compromising the antipathogenic activity of the compound. Because silvestrol exhibits immune-modulatory potential [24], we speculated that this effect is a consequence of elF4A inhibition. Other rocaglates such as CR-31-B (−) and zotatifin possibly interact with the host immune system in a similar manner, thereby bolstering the antipathogenic effect and/or promoting resolution of inflammation and tissue damage.

Infections activate the innate immune system, which is mainly driven by dendritic cells and macrophages. These mononuclear phagocytes release chemokines and cytokines to recruit and stimulate immune cells of the adaptive immune system, such as B cells and T cells. B cells differentiate into plasma cells, releasing antibodies that mark the viruses and promote their clearance. T cells impact viral infections by virtue of their capacity to combat intracellular pathogens. The effector functions of T cells are mediated through cytokines/chemokines and by the direct cytotoxicity of infected cells. However, specific subsets of immune cells, including regulatory T cells, Th2 cells and M2 macrophages, dampen antiviral responses and initiate inflammation resolution. These anti-inflammatory functions of immune cells are essential to preventing tissue damage.

In the present study, we addressed the question whether the immune modulatory properties of silvestrol and other rocaglates are compound-specific effects or whether they are mediated via the inhibition of elF4A. For this purpose, we investigated and compared the immunomodulatory effects of three rocaglates (silvestrol, zotatifin and CR-31-B (−)) on primary human monocyte-derived macrophages (MdMs), monocyte-derived dendritic cells (MdDCs), T cells and B cells. To differentiate between compound-specific and target-specific effects, the inactive enantiomer CR-31-B (+) was analyzed. The impact of rocaglates on cell viability, cell-type-specific surface markers, released cytokines and energy metabolism during differentiation and polarization/activation of different human immune cells was investigated.

## 2. Results

### 2.1. Rocaglates Affected the Differentiation and Polarization of MdMs

**Figure 1:**
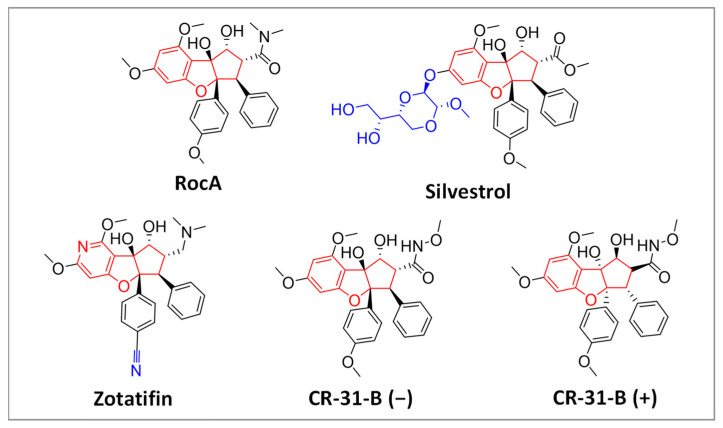
Chemical structure of natural (upper row) and synthetic (lower row) rocaglates. The natural compounds RocA and silvestrol were extracted from plants of the genus *Aglaia*. Rocaglates share a typical central cyclopenta[b]benzofurane moiety. The synthetic rocaglate CR-31-B consists of a racemic mixture of two enantiomers, of which the (−)-enantiomer is biologically active while the (+)-enantiomer is inactive.

Recently, we observed immune modulatory effects for the natural rocaglate silvestrol in monocyte-derived macrophages (MdMs) as well as in M1- and M2-MdMs [24]. To clarify whether the observed immune modulatory effects are drug- or target-specific, we investigated whether the synthetic rocaglates zotatifin and CR-31-B (−) reveal similar immune-modulatory effects. CR-31-B (+), which is the inactive enantiomer of CR-31-B (−) and cannot bind in the rocaglate binding pocket of the eIF4A-RNA complex (see also Supplementary Figures S1 and S2), served as a negative control in our experiments. (The chemical structures of the respective rocaglates are shown in Figure 1).

**Figure 2:**
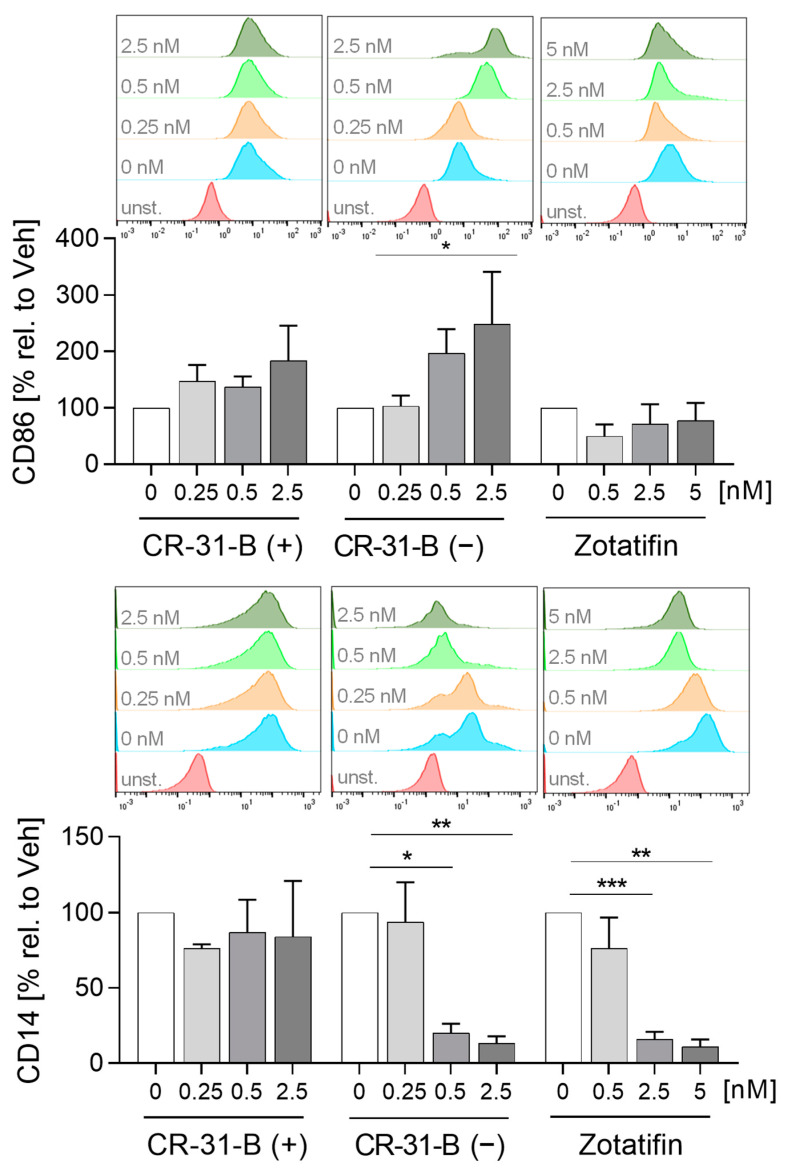
Rocaglates influenced macrophage surface marker expression. Primary human monocytes were stimulated for 7 days (MdMs) with GM-CSF (10 ng/mL) in the presence or absence of the rocaglates. Surface marker expression of MdMs was measured with a MACSQuant^®^ Analyzer 10. The geometric mean of the fluorescence intensity was related to the vehicle control. Above the graphs, representative curves of the geometric mean fluorescence intensity are shown. *N* = 3–5. For statistical analysis, mixed-effect analysis with Dunnett’s multiple comparisons test was used. * *p* < 0.05, ** *p* < 0.01, and *** *p* < 0.001 indicate significant differences between rocaglate- and vehicle-treated samples. Abb.: unst., unstained.

**Figure 3:**
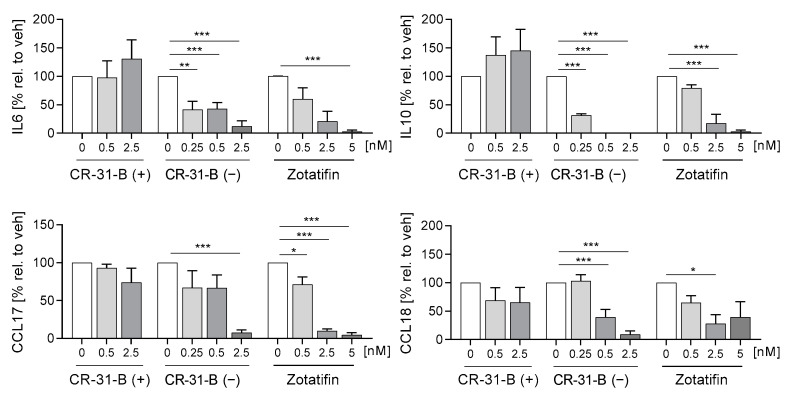
Rocaglates influenced the cytokines released by macrophages. Primary human monocytes were stimulated for 7 days (MdMs) with GM-CSF (10 ng/mL) in the presence or absence of the rocaglates. Released cytokines were measured with a cytometric bead array or ELISA. *N* = 3–5. For statistical analysis, mixed-effect analysis with Dunnett’s multiple comparisons test was used. * *p* < 0.05, ** *p* < 0.01, and *** *p* < 0.001 indicate significant differences between rocaglate- and vehicle-treated samples.

First, we tested whether the inhibition of elF4A influenced the differentiation of monocytes to MdMs. We analyzed several inflammatory surface markers including CD14, CD80, CD86, CD163, CD206, HLA-DR and TREM2; and cytokines including IL6, IL10, CCL17 and CCL18. CR-31-B (−) increased the surface marker expression of CD86, HLA-DR and CD163 while decreasing CD14 levels. CR-31-B (+) induced the expression of CD206, while zotatifin reduced the expression of CD14, CD206 and CD80 (Figure 2, Supplementary Figure S3, Table 1). Interestingly, all rocaglates (including silvestrol—published data [24]), but not CR-31-B (+), decreased the release of IL6, IL10, CCL17 and CCL18 as well as the expression of CD14 (Figure 3, Table 1), indicating that rocaglates are likely to suppress recruitment of immune cells and the resulting immune response.

**Figure 4:**
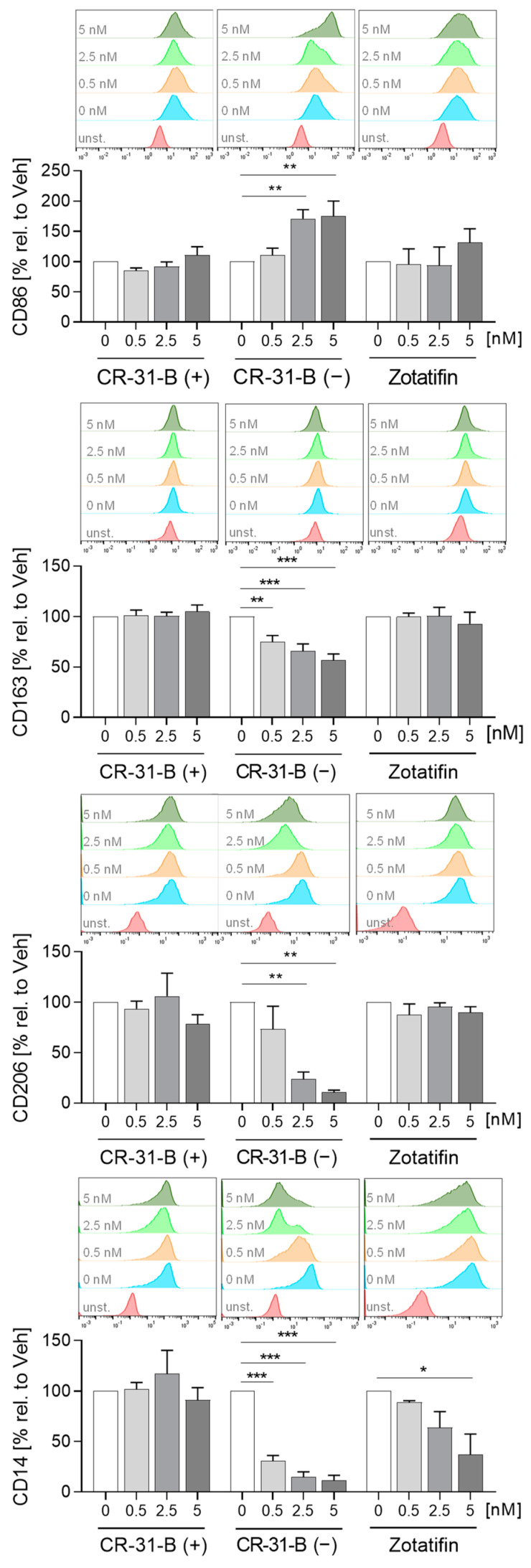
Rocaglates affected the functions of M1 polarized macrophages. Primary human monocytes differentiated to MdMs were polarized to M1 MdMs in the presence or absence of rocaglates. Surface marker expression of M1 MdMs was measured with a MACSQuant^®^ Analyzer 10. The geometric mean of the fluorescence intensity was related to vehicle control. Above the graphs, a representative histogram of the geometric mean fluorescence intensity is shown. *N* = 3–6. For statistical analysis, mixed-effect analysis with Dunnett’s multiple comparisons test was used. * *p* < 0.05, ** *p* < 0.01, and *** *p* < 0.001 indicate significant differences between rocaglate- and vehicle-treated samples. Abb.: unst., unstained.

Next, we investigated whether the inhibition of elF4A influences the polarization of MdMs to M1 or M2 MdMs. CR-31-B (−) increased the expression of CD86 and decreased that of CD14, CD206 and CD163 in M1 MdMs, CR-31-B (+) had no effect on surface marker expression in M1 MdMs and zotatifin reduced the expression of CD14 and TREM2 (Figure 4, Supplementary Figure S4A, Table 2). The release of CCL18, CCL2 and IL8 was reduced in CR-31-B (−)-treated M1 MdMs. CR-31-B (+) increased the release of CCL17 in M1 MdMs. Surprisingly, zotatifin did not affect the release of any cytokine from M1 MdMs (Supplementary Figures S4B and S5, Table 2).

**Figure 5:**
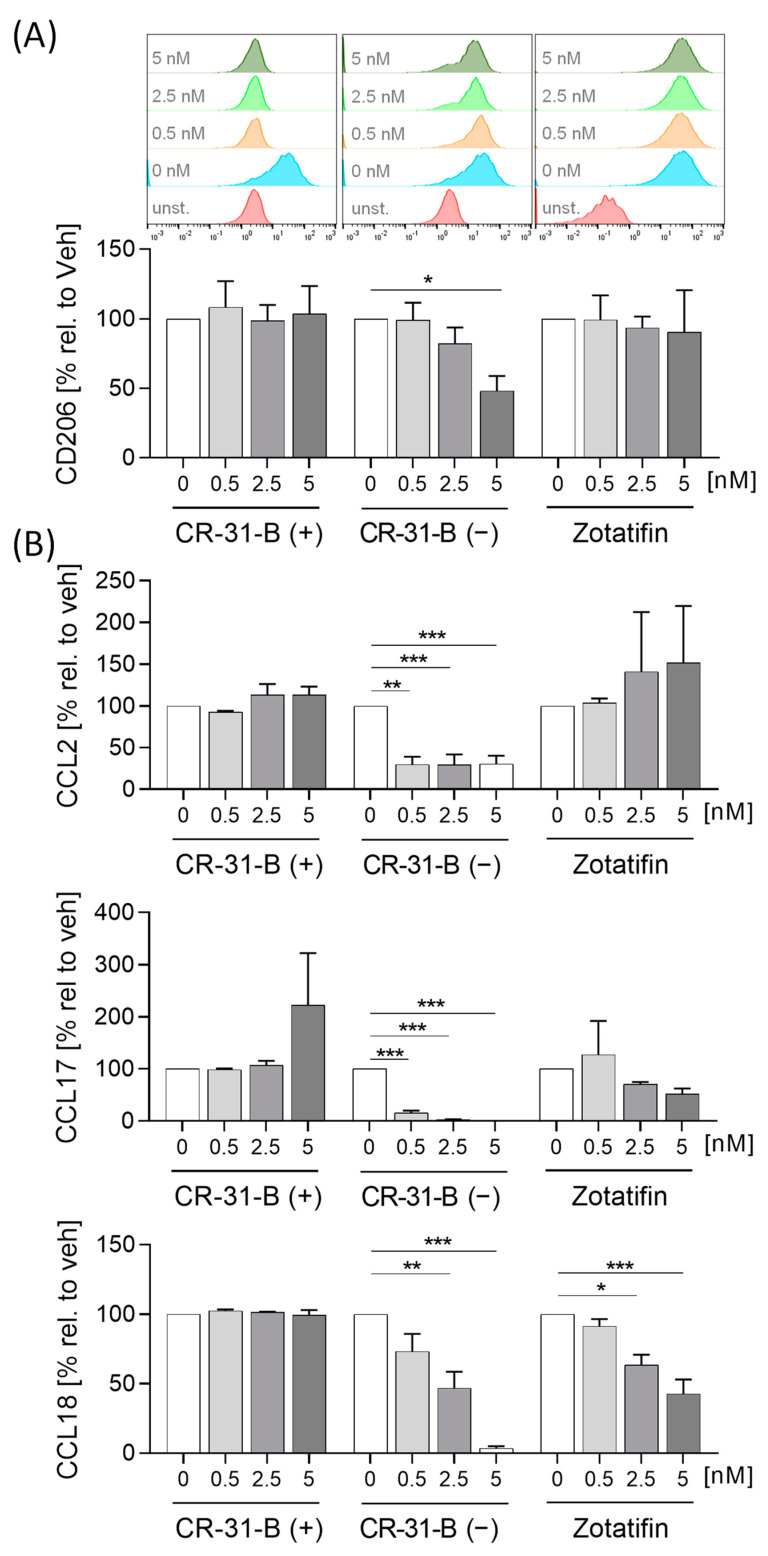
Rocaglates affected the functions of M2 polarized macrophages. Primary human monocytes differentiated to MdMs were polarized to M2 MdMs in the presence or absence of rocaglates. (**A**) Surface marker expression of M2 MdMs was measured with a MACSQuant^®^ Analyzer 10. The geometric mean of the fluorescence intensity was related to vehicle control. Above the graphs, a representative histogram of the geometric mean fluorescence intensity is shown. (**B**) Released cytokines in the supernatant of M2 MdMs were measured with cytometric bead array or ELISA. *N* = 3–6. For statistical analysis, mixed-effect analysis with Dunnett’s multiple comparisons test was used. * *p* < 0.05, ** *p* < 0.01, and *** *p* < 0.001 indicate significant differences between rocaglate- and vehicle-treated samples. Abb.: unst., unstained.

In M2 MdMs, the effects on surface marker expression of any rocaglate treatment were rather low. CR-31-B (−) reduced the expression of CD206, while zotatifin increased the expression of CD86 (Figure 5A, Supplementary Figure S6, Table 3). Zotatifin reduced the release of IL10 and CCL18 while CR-31-B (−) also reduced the release of several cytokines (CCL17, CCL18, CCL2) and increased that of TNFα (Figure 5B, Supplementary Figure S7, Table 3).

These data indicate that rocaglates have ambiguous effects on surface marker expression in MdMs and M1/M2 MdMs, whereas they generally reduce the cytokine release by these cell types, apart from zotatifin on polarizing M1 macrophages.

### 2.2. Rocaglates Affected the Differentiation and Activation of MdDCs

**Figure 6:**
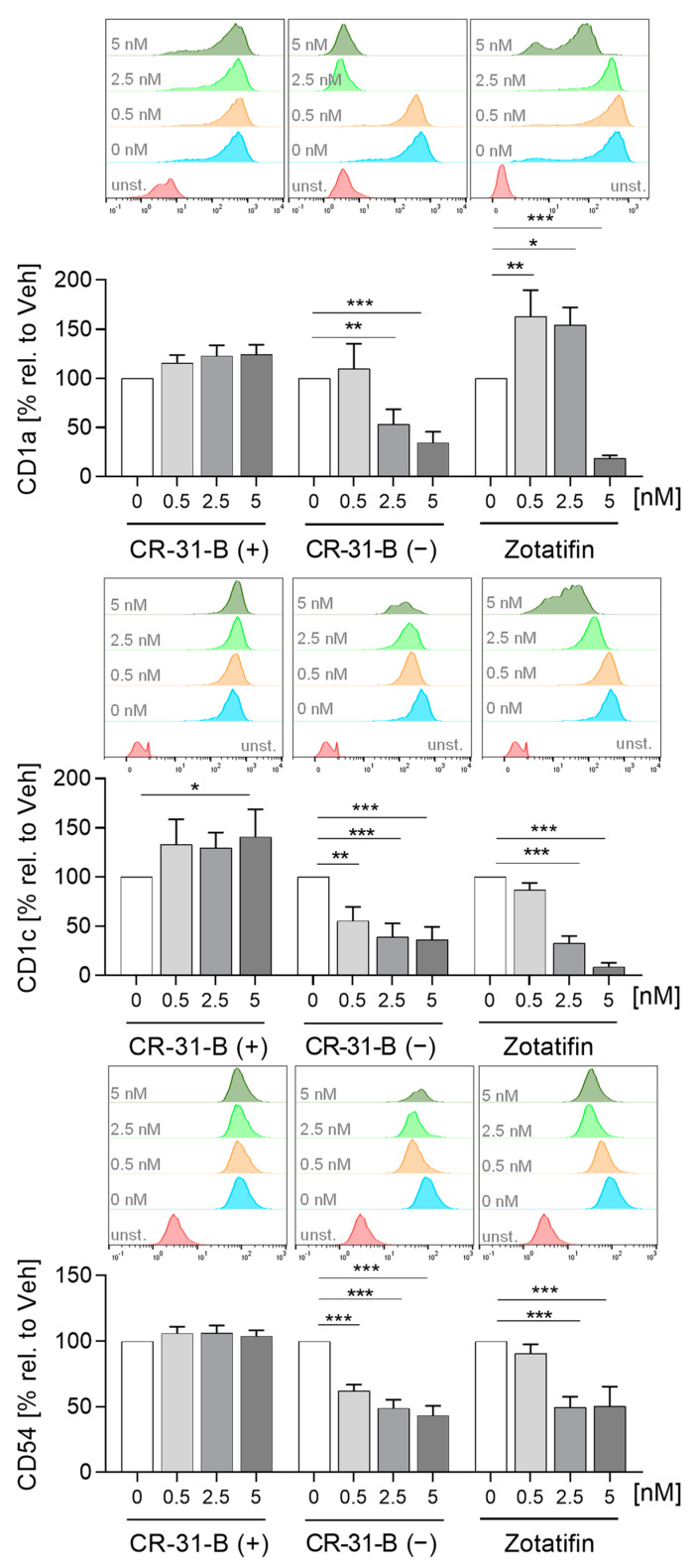
Rocaglates influenced the surface marker expression of MdDCs. Primary human monocytes were differentiated to MdDCs for 5 days with GM-CSF (10 ng/mL) and IL-4 (10 ng/mL) in the presence or absence of rocaglates. Surface marker expression was measured with a MACSQuant^®^ Analyzer 10. The geometric mean of the fluorescence intensity was related to vehicle control. Above the graphs, a representative histogram of the geometric mean fluorescence intensity is shown. *N* = 3–6. For statistical analysis, mixed-effect analysis with Dunnett’s multiple comparisons test was used. * *p* < 0.05, ** *p* < 0.01, and *** *p* < 0.001 indicate significant differences between rocaglate- and vehicle-treated samples. Abb.: unst., unstained.

**Figure 7:**
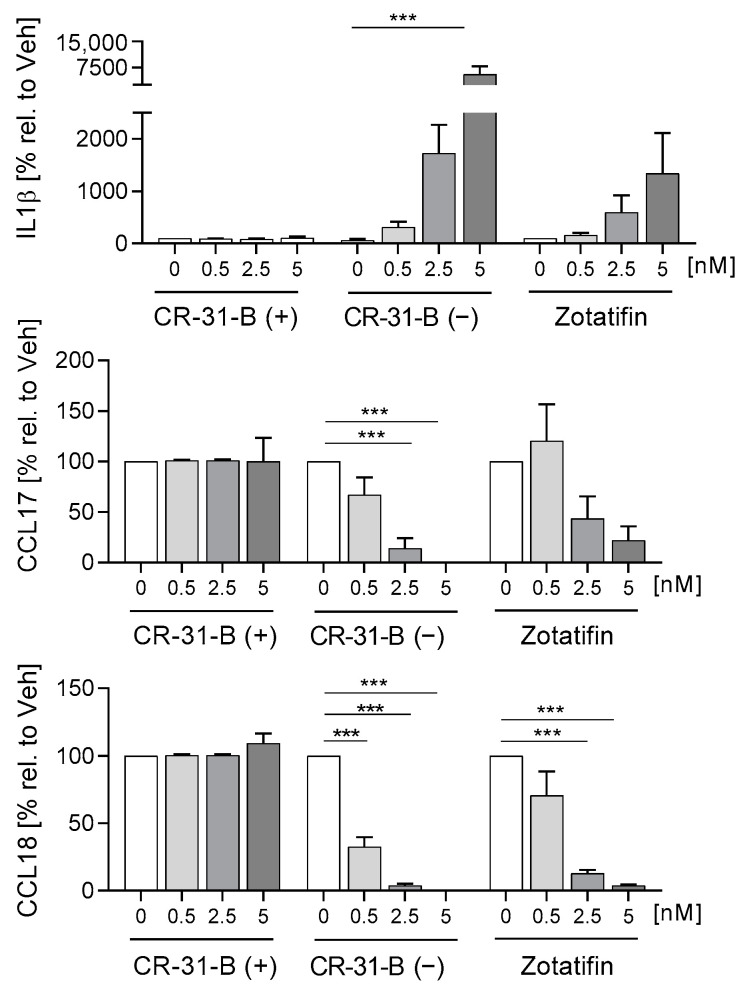
Rocaglates influenced the cytokines released by MdDCs. Primary human monocytes were differentiated to MdDCs for 5 days with GM-CSF (10 ng/mL) and IL-4 (10 ng/mL) in the presence or absence of rocaglates. Released cytokines were measured with a cytometric bead array. *N* = 3–6. For statistical analysis, mixed-effect analysis with Dunnett’s multiple comparisons test was used. *** *p* < 0.001 indicate significant differences between rocaglate- and vehicle-treated samples.

To analyze the effect of rocaglates on the differentiation of monocyte derived dendritic cells (MdDCs), primary monocytes were differentiated in the presence or absence of rocaglates to MdDCs. CR-31-B (−) reduced the expression of several markers (CD1a, CD1c, CD54, HLA-DR, CD40, CD80, CD209, CD86) and increased CD83 and CD141. Zotatifin reduced CD1c, CD54, CD206 and CD209 and increased CD1a (at low concentrations) and CD141 in MdDCs. CR-31-B (+) only increased CD1c (Figure 6, Supplementary Figures S8 and S9A, Table 4). CR-31-B (−) reduced the release of chemokines (CCL17, CCL18) and increased IL-1β in MdDCs, whereas zotatifin reduced CCL18 and IL10 (Figure 7, Supplementary Figure S9B, Table 4).

Interestingly, only the upregulation of CD141, the reduced expression of CD54 and CD209 and the release of CCL18 were observed in the presence of all three rocaglates. The negative control CR-31-B (+) showed no effect at all. The reduction of CD209 may potentially influence viral defense mechanisms since several viruses, e.g., dengue viruses, use CD209, a C-type lectin, to infect dendritic cells [25].

**Figure 8:**
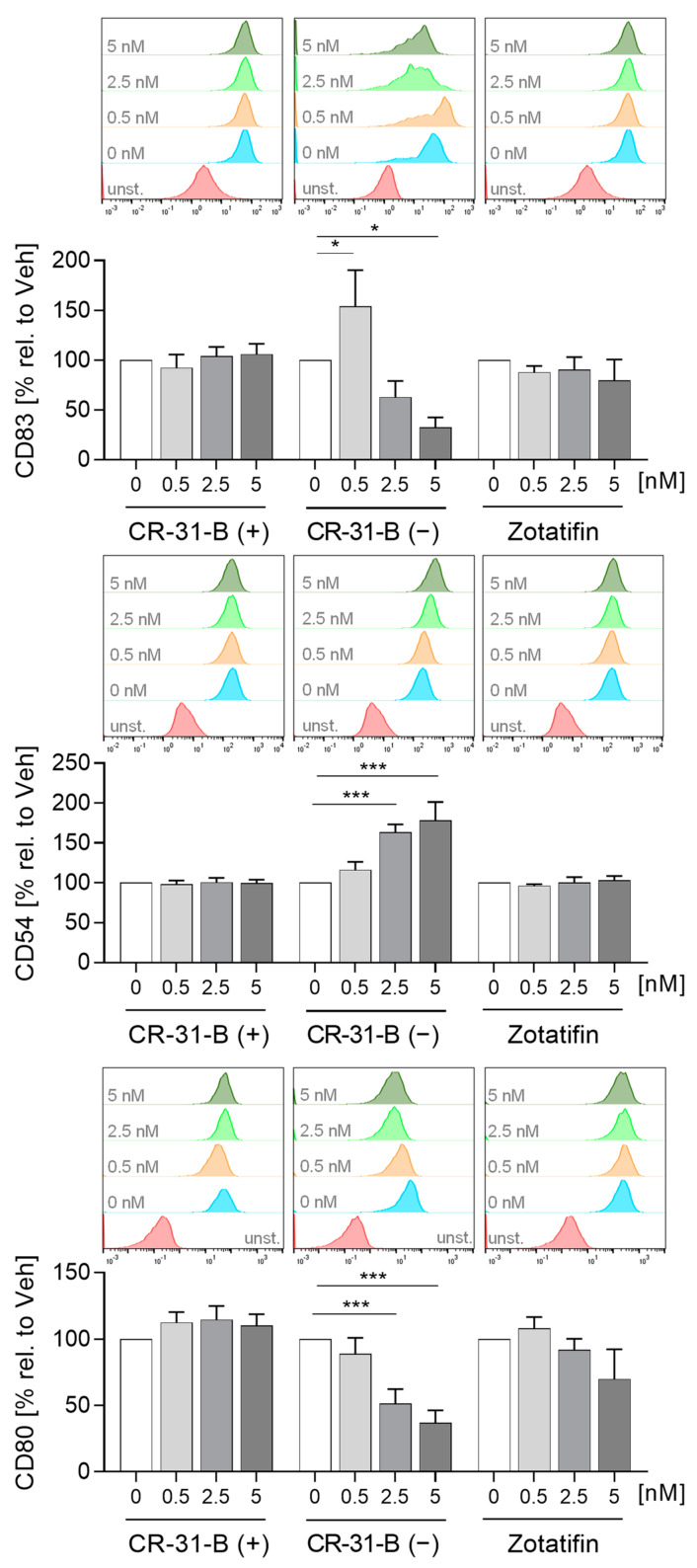
Rocaglates influenced the surface marker expression of activated MdDCs. MdDCs were activated with a cytokine mixture in the presence or absence of rocaglates. Surface marker expression was measured with a MACSQuant^®^ Analyzer 10. The geometric mean of the fluorescence intensity was related to vehicle control. Above the graphs, a representative histogram of the geometric mean fluorescence intensity is shown. *N* = 3–6. For statistical analysis, mixed-effect analysis with Dunnett’s multiple comparisons test was used. * *p* < 0.05, and *** *p* < 0.001 indicate significant differences between rocaglate- and vehicle-treated samples. Abb.: unst., unstained.

**Figure 9:**
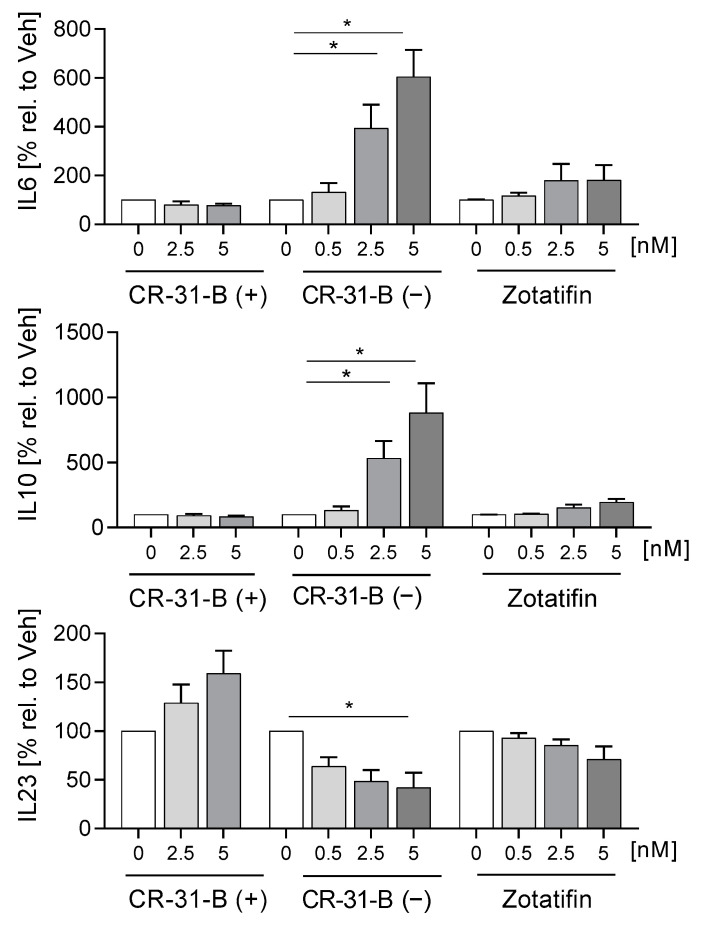
Rocaglates influenced the cytokines released by activated of MdDCs. MdDCs were activated with a cytokine mixture in the presence or absence of rocaglates. Released cytokines were measured with a cytometric bead array. *N* = 3–6. For statistical analysis, mixed-effect analysis with Dunnett’s multiple comparisons test was used. * *p* < 0.05 indicate significant differences between rocaglate- and vehicle-treated samples.

To determine whether rocaglates affect the activation of MdDCs, the MdDCs were activated with a mixture of IL6, IL1β, TNFα and PGE_2_ in the presence or absence of the rocaglates. CR-31-B (−) increased the expression of several surface markers (CD1a, CD1c, CD54, CD83 (at low concentrations)) and reduced that of HLA-DR and CD80, whereas zotatifin increased only the expression of CD1a at 2.5 nM. CR-31-B (+) had no effect on the surface marker expression profile at all (Figure 8, Supplementary Figures S10 and S11, Table 5). Moreover, CR-31-B (−) reduced CCL17, IL8 and IL23 and increased IL6 and IL10, while zotatifin and CR-31-B (+) did not affect cytokine release (Figure 9, Supplementary Figure S12A, Table 5). Only silvestrol (published data [24]) and CR-31-B (−) had a common effect on cytokine regulation (upregulation of IL6 and IL10 and downregulation of IL8 and IL23).

### 2.3. Rocaglates Suppressed Energy Metabolism

To meet their energy requirements, M1 macrophages predominantly use glycolysis as an energy source, whereas the main energy source of M2 macrophages is oxidative phosphorylation [26]. Since the rocaglates influence the immune status of the cells, we investigated whether they also influence the energy metabolism in these cell types. We determined the extracellular acidification rate (ECAR) as a marker for glycolysis and oxygen consumption rate (OCR) as a marker for mitochondrial respiration. The OCR determined for M1 macrophages was about 150 pmol/min, for M2 MdMs about 40 pmol/min and for activated MdDCs about 18 pmol/min, whereas the ECAR was 14 mpH/min, 4 mpH/min and 2 mpH/min for M1 MdMs, M2 MdMs and activated MdDCs, respectively. These data confirm that M1 MdMs have the highest energy requirements and that they predominantly use glycolysis.

**Figure 10:**
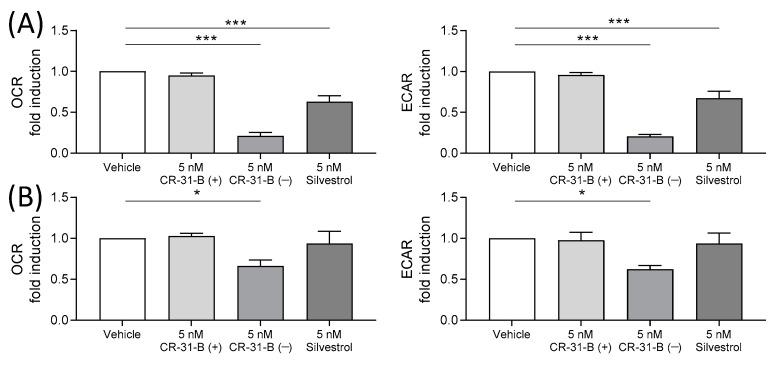
Rocaglates impaired the energy metabolism of M1 and M2 MdMs. MdMs were polarized to M1 (**A**) or M2 (**B**) MdMs in the presence or absence of rocaglates. Oxygen consumption rate (OCR) and extracellular acidification rate (ECAR) were measured with the Seahorse XFe96 analyzer (Agilent, Waldbronn, Germany) over a total time period of 160 min. The OCR and ECAR values after 60 min of rocaglate treatment were related to the vehicle-treated samples to obtain fold induction. *N* = 4. For statistical analysis, one-way ANOVA with Dunnett’s multiple comparisons test was used. * *p* < 0.05 and *** *p* < 0.001 show significant differences between rocaglate and vehicle treatment.

CR-31-B (−) and silvestrol led to a significant decrease in the ECAR and OCR levels in M1 MdMs (Figure 10A). In M2 MdMs, only CR-31-B (−) was able to reduce the ECAR and OCR levels (Figure 10B). In activated MdDCs, CR-31-B (−) and silvestrol did not influence energy metabolism (Supplementary Figure S12B).

Taken together, the results show that the energy metabolism of cell types with a high energy requirement (M1 MdMs) are influenced more by rocaglates than they are by cell types with a low energy requirement (activated MdDCs).

### 2.4. CR-31-B (−) and Zotatifin Reduced T-Cell Proliferation and Cytokine Release

**Figure 11:**
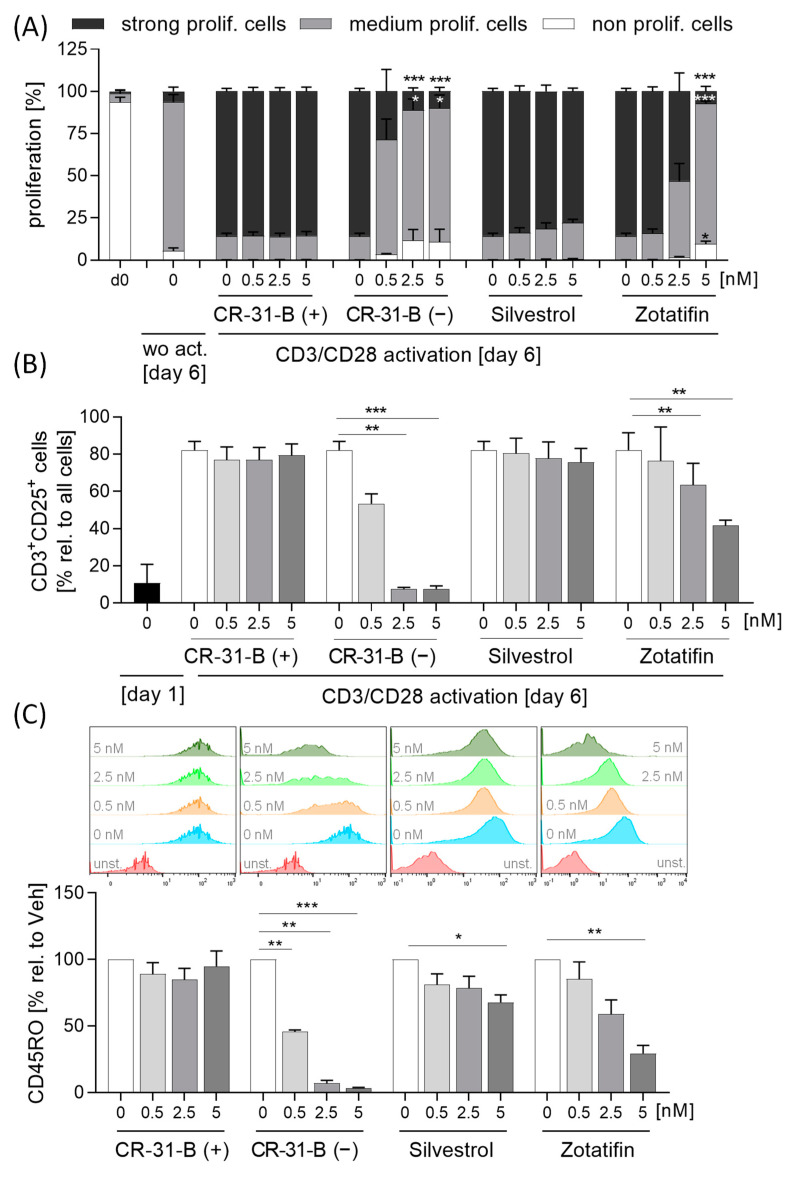
Rocaglates impaired T-cell activation. Primary human T cells were activated with anti-CD3/anti-CD28 for 5 days in the presence or absence of rocaglates. (**A**) Proliferation was detected with CellTrace^TM^ violet and flow cytometry. The rocaglate-treated samples were related to the vehicle-treated samples. (**B**,**C**) CD3+CD25+ cells (**B**) and the surface marker CD45RO (**C**) on CD3+CD25+ cells were determined using flow cytometry. Above the graphs, a representative histogram of the geometric mean fluorescence intensity is shown. *N* = 4. For statistical analysis, two-way ANOVA with Dunnett’s multiple comparisons test was used. * *p* < 0.05, ** *p* < 0.01, and *** *p* < 0.001 show significant differences between rocaglate and vehicle treatment. Abb.: unst., unstained.

To investigate whether, in addition to the those of the innate immune system, cells of the acquired immune system are also influenced by the inhibition of elF4A, we activated T cells with anti-CD3 and anti-CD28 and determined the proliferation rate, surface marker expression (CD69, CD154, CD134 and CD25) and cytokine release (IL17A, IL10, IL4 and IFNγ). Interestingly, CR-31-B (−) and zotatifin inhibited the proliferation of T cells, whereas silvestrol had no influence on the proliferation rate (Figure 11A).

**Figure 12:**
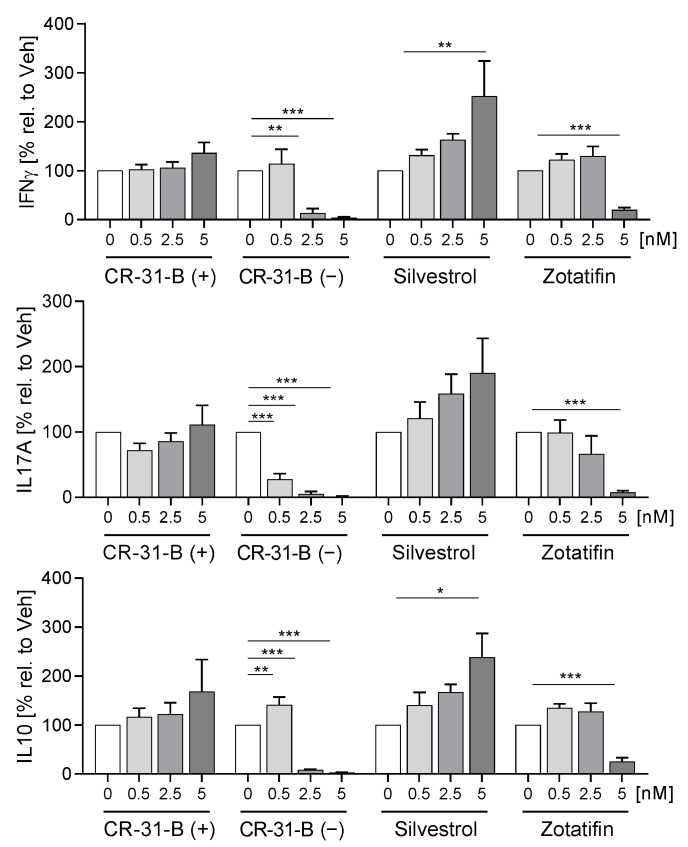
Rocaglates influence cytokine release of activated T cells. Primary human T cells were activated with anti-CD3/anti-CD28 for 5 days in the presence or absence of rocaglates. Released cytokines were determined with a cytometric bead array; *N* = 4. For statistical analysis, two-way ANOVA with Dunnett’s multiple comparisons test was used. * *p* < 0.05, ** *p* < 0.01, and *** *p* < 0.001 show significant differences between rocaglate and vehicle treatment.

Only CR-31-B (−) induced apoptosis in T cells (Supplementary Figure S13A). During the activation of T cells, the number of CD25-positive cells increased, while CR-31-B (−) and zotatifin reduced the number of CD25-positive T cells (Figure 11B). Moreover, CR-31-B (−) reduced the expression of CD134 and CD45RO, zotatifin reduced that of CD45RO and increased that of CD69 and CD154, and silvestrol reduced that of CD45RO and CR-31-B (+) but had no effects on the surface marker expression profile of activated T cells (Figure 11C, Table 6, Supplementary Figure S13B). The activation of T cells with anti-CD3 and anti-CD28 led to an increase in IL10, IL17 and IFNγ, while IL4 was not detectable. Interestingly, CR-31-B (−) at 2.5 nM and zotatifin at 5 nM reduced all detectable cytokines, whereas silvestrol increased IFNγ and IL10 and CR-31-B (+) had no effect (Figure 12, Table 6). These data indicate that predominantly CR-31-B (−) and zotatifin, the rocaglates without a dioxan moiety prevent the activation of T cells by reducing cytokine release and proliferation.

### 2.5. Rocaglates Prevented Activation of B Cells

**Figure 13:**
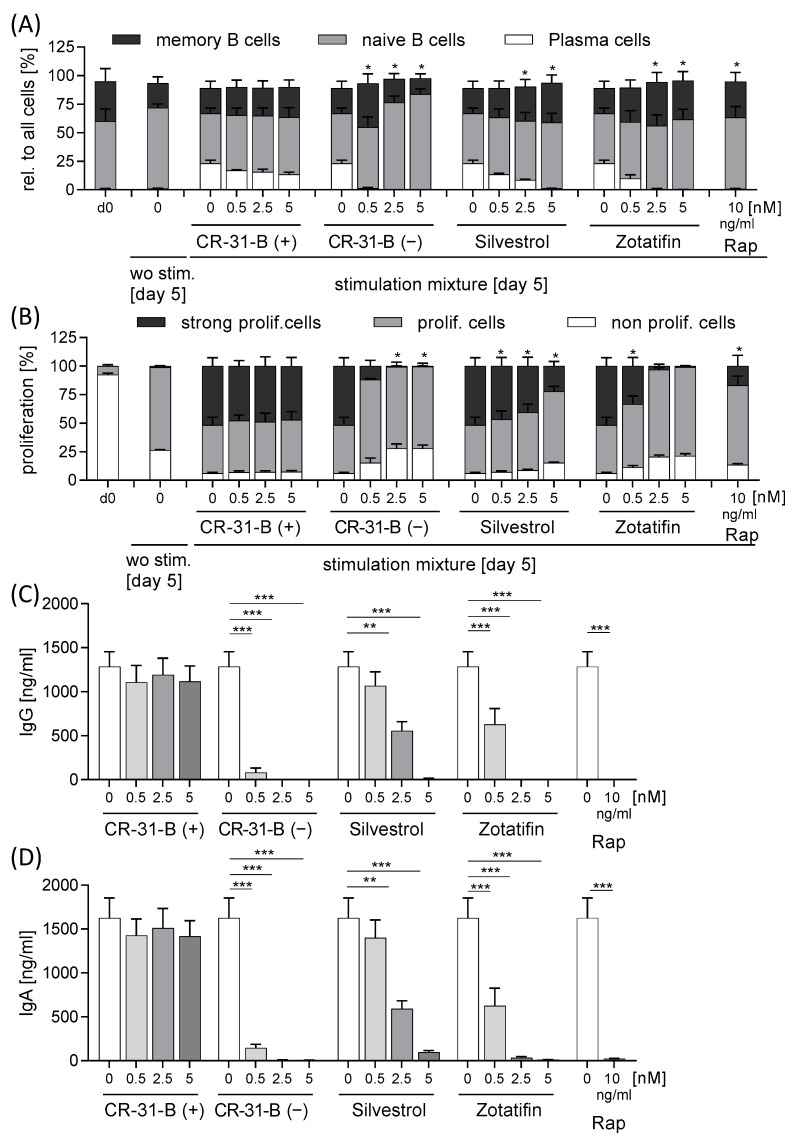
Rocaglates prevented B-cell activation. Primary human B cells were activated with a mixture of IL21, sCD40L, CpG and anti-IgM for 5 days in the presence or absence of rocaglates. (**A**) Distribution of memory B cells, naïve B cells and plasma B cells was determined with flow cytometry. (**B**) Proliferation was detected with CellTrace^TM^ violet with flow cytometry. The rocaglate-treated samples were related to the vehicle-treated samples. (**C**,**D**) Released immune globulins were determined with ELISA. *N* = 3. For statistical analysis, one-way ANOVA with Dunnett’s multiple comparisons test was used. * *p* < 0.05, ** *p* < 0.01, and *** *p* < 0.001 show significant differences between rocaglate and vehicle treatment.

To analyze the effect of rocaglates on B cells, we activated these cells with a mixture of IL21, sCD40L, CpG and anti-IgM for 5 days in the presence or absence of the rocaglates. The activation led to the proliferation of B cells, formation of plasma cells and the release of immune globulin (Ig)G and IgA. As a negative control, we used rapamycin, which inhibits these processes. Interestingly, all three rocaglates reduced B-cell proliferation, formation of plasma cells and the release of IgG and IgA, whereas silvestrol showed the least pronounced effects (Figure 13, Supplementary Figure S14, Table 7).

### 2.6. Rocaglates Prevented Activation of T Cells in Coculture with Dendritic Cells

Our data suggest that the inhibition of elF4A affects dendritic cell (DC) antigen presentation and T-cell activation (reducing HLA-DR and CD40); therefore, in a DC/T-cell coculture model, we analyzed the effect of rocaglate-treated DCs on T-cell activation. For this purpose, we used autologous DCs and T cells. DCs were pretreated with rocaglates and stimulated for 24 h with a cytokine mixture. The rocaglates did not induce apoptosis in DCs (Supplementary Figure S15A, Table 8). CR-31-B (−) reduced several surface markers (CD40, HLA-DR and CD86) and increased CD83. Silvestrol increased CD83 and CD40, whereas zotatifin increased only CD83 (Supplementary Figure S15B, Table 8).

**Figure 14:**
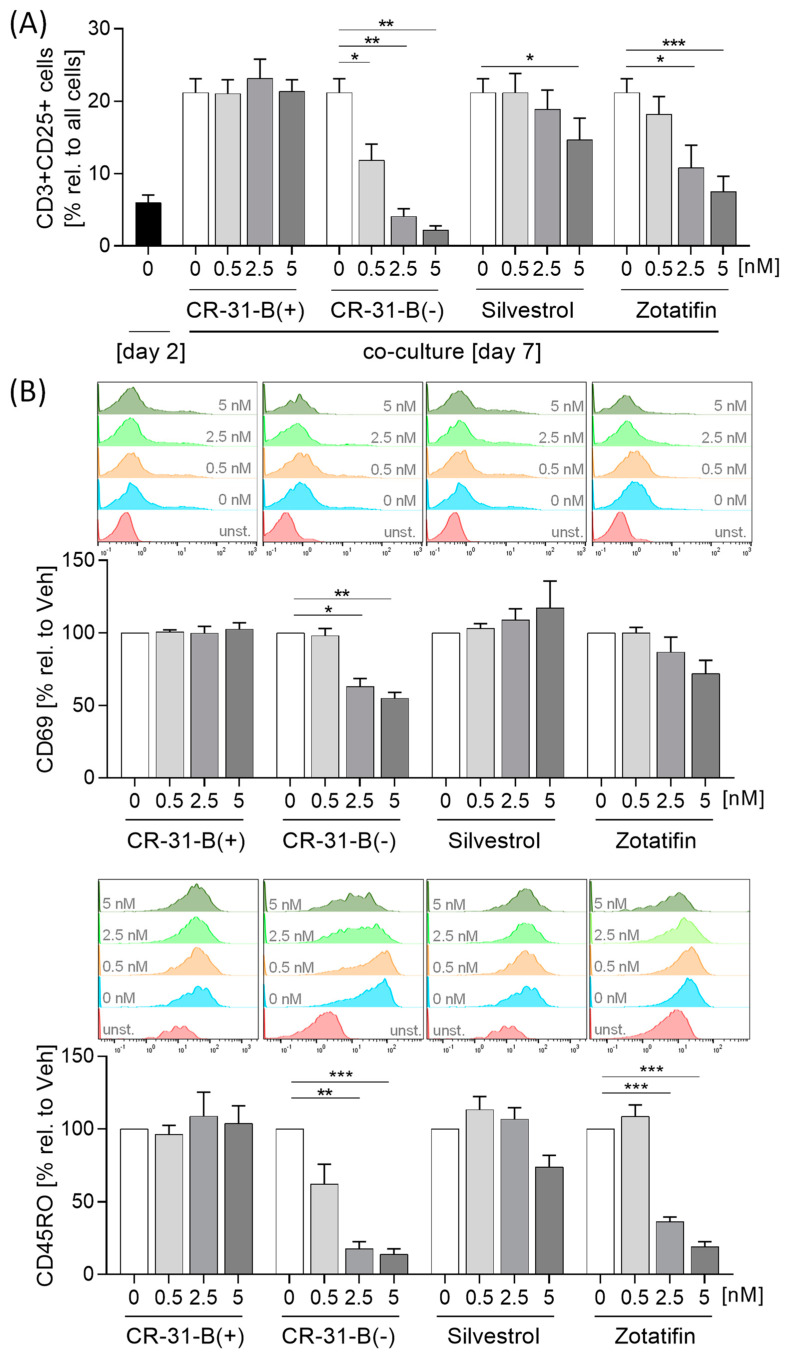
Rocaglates prevent DC-induced T-cell activation. DCs were activated with a mixture of inflammatory mediators (IL6, TNFα, IL1β and PGE_2_) for 24 h in the presence or absence of rocaglates and then coincubated with homologous T cells for 5 days. (**A**) CD3+CD25+ cells and (**B**) surface marker CD69 and CD45RO on CD3+CD25+ cells were determined with flow cytometry. Above the graphs, a representative histogram of the geometric mean fluorescence intensity is shown. *N* = 4. For statistical analysis, one-way ANOVA with Dunnett’s multiple comparisons test was used. * *p* < 0.05, ** *p* < 0.01, and *** *p* < 0.001 show significant differences between rocaglate and vehicle treatment. Abb.: unst., unstained.

**Figure 15:**
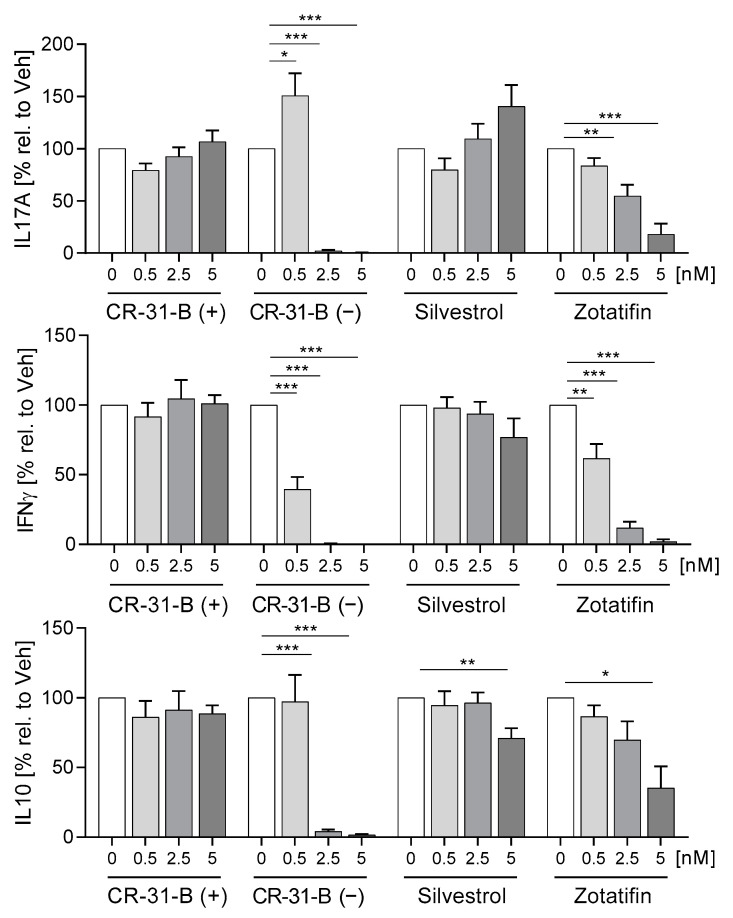
Rocaglates influence DC-induced cytokine release of T cells. DCs were activated with a mixture of inflammatory mediators (IL6, TNFα, IL1β and PGE_2_) for 24 h in the presence or absence of rocaglates and then coincubated with homologous T cells for 5 days. Released cytokines were determined with cytometric bead array. *N* = 4. For statistical analysis, one-way ANOVA with Dunnett’s multiple comparisons test was used. * *p* < 0.05, ** *p* < 0.01, and *** *p* < 0.001 show significant differences between rocaglate and vehicle treatment.

The pretreated DCs were cocultured with T cells for 5 days, and 2.5 nM of CR-31-B (−) induced apoptosis of DCs and T cells after 5 days of coculture (Supplementary Figures S16A and S17A). Interestingly, CR-31-B (−) and zotatifin increased several DC surface markers (CD83, CD80 and CD40), and CR-31-B (−) additionally increased HLA-DR under these coculture conditions. With silvestrol and CR-31-B (+), no effects were observed (Supplementary Figure S17B). CR-31-B (−) and zotatifin reduced T-cell proliferation, whereas silvestrol and CR-31-B (+) had no effect (Supplementary Figure S16B). All three rocaglates reduced the number of CD25^+^CD3^+^ cells (Figure 14A). CR-31-B (−) reduced T-cell surface markers (CD69, CD45RO, CD154); zotatifin reduced CD45RO and CD154, whereas silvestrol and CR-31-B (+) had no effect on the T-cell surface marker profile (Figure 14B, Supplementary Figure S16C, Table 9). CR-31-B (−) and zotatifin reduced the release of IL17A, IFNγ and IL10, whereas silvestrol only decreased IL10, and CR-31-B (+) had no effect (Figure 15). These data indicate that the inhibition of elF4A in DCs by rocaglates generally leads to the reduced activation of T cells, with silvestrol appearing to be the least active.

## 3. Discussion

**Figure 16:**
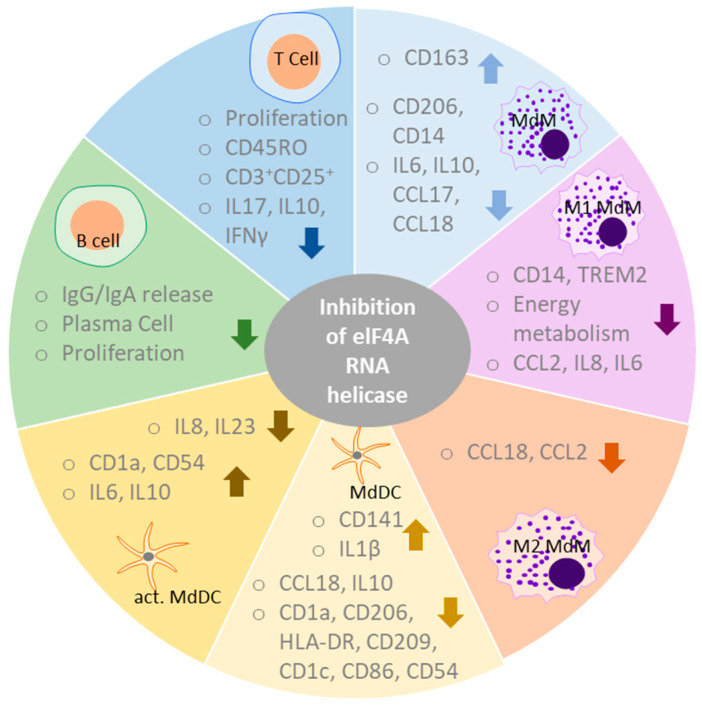
Overview of effects on immune cell function due to the inhibition of elF4A. The overview depicts results shown by at least two of the rocaglates and not observed for the inactive enantiomer CR-31-B (+). Rocaglates impaired the differentiation of macrophages by reducing surface marker expression and cytokine release. They reduced the activation of M1 MdMs as demonstrated by decreasing surface marker levels, cytokine release and energy metabolism. In M2 MdMs, rocaglates also decreased cytokine release. Rocaglates impaired the differentiation of monocytes to dendritic cells as shown indicated by the regulation of several surface markers and cytokines. In activated DCs, rocaglates led to a modulation of cytokine release and surface marker expression. Rocaglates reduced T-cell activation as indicated by a decrease in proliferation, activation, cytokine release and the expression of CD45RO. Rocaglates further impaired B-cell activation as indicated by reduced proliferation, plasma cell formation and IgG/IgA release.

We studied the effects of rocaglates (silvestrol, CR-31-B (−) and zotatifin) on immune cell functions. To determine whether the observed effects could be attributed to elF4A inhibition, CR-31-B (+), the inactive enantiomer of CR-31-B (−), was used as a negative control in all experiments. Effects seen with at least two of the inhibitors and that were not observed with CR-31-B (+) were considered to be the potential effects mediated via the inhibition of elF4A (Figure 16).

Targeting elF4A with rocaglates impaired the differentiation of macrophages by reducing surface marker expression (CD14, CD206) and cytokine release (IL6, IL10, CCL17, CCL18). Moreover, the inhibition of elF4A reduced surface markers (CD14, TREM2), cytokine release (CCL2, IL8, IL6) and energy metabolism in M1 MdMs while reducing cytokine release (CCL18, CCL2) in M2 MdMs. For the differentiation of monocytes to dendritic cells, elF4A seems to play an important role since rocaglate treatment exhibited marked effects on differentiation as shown by the reduction of several surface markers (CD1a, CD1c, HLA-DR, CD54, CD86, CD206 and CD209), decrease in cytokine release (CCL18, IL10) and an increase in CD143 and IL1β. In activated DCs, the inhibitors of elF4A led to an increase in IL8 and IL23 but a decrease in IL6, IL10 and the surface marker CD1a and CD54. Moreover, rocaglates reduced proliferation, activation, cytokine release (IL17, IL10, IFNγ) and the expression of CD45RO in T cells. In B cells, pharmacological inhibition of elF4A was accompanied by reduced proliferation, plasma cell formation and IgG/IgA release. Overall, the inhibition of elF4A seems to be associated with the downregulation of the immune response.

Inflammatory stimuli can induce a change in the energy metabolism of the immune cells by switching from oxidative phosphorylation to glycolysis in order to provide the necessary connections for the cell in this exceptional “inflammatory” situation. This mechanism, which is similar to the Warburg effect, is known as an essential immune response named immune metabolism [27]. CR-31-B (−) and silvestrol reduced metabolic activity (glycolysis, oxidative phosphorylation) in M1 and/or M2 macrophages. Chen et al. recently also reported that CR-31-B (−) reduces both oxidative phosphorylation and glycolysis in pancreatic ductal adenocarcinoma cells. They also observed that CR-31-B (−) reduces glucose uptake and the expression of the glucose transporter SLC2A6 (GLUT6) [28]. Therefore, intracellular glucose level may decrease as a result of the downregulation of the glucose transporter, and the cell is forced to limit energy production via glycolysis and oxidative phosphorylation. However, Maedera et al. showed that GLUT6 is expressed in murine macrophages in the lysosomal membrane [29] and not in the plasma membrane and thus cannot be responsible for glucose uptake. They also observed that lipopolysaccharide regulates the expression of GLUT6 via NF-κB. In this context, it is worth noting that rocaglates have been reported to inhibit NF-κB [30]. Thus, our results and the results of Chan et al. and Maedera et al. suggest that CR31-B (−) and silvestrol may reduce GLUT6 expression via the inhibition of NF-κB, thereby impairing energy metabolism and suppressing the immune response in M1 macrophages. However, this could not be confirmed in murine M1 macrophages since the lack of GLUT6 did not affect the LPS-induced immune reaction with regard to cytokine release and glycolysis [31]. In the case of M2 macrophages, we did not observe a pronounced effect on immune responses. In addition, the effect on energy metabolism was less than that of M1 macrophages. This is likely to be due to the fact that M2 macrophages do not express GLUT6 [31]. The role of NF-κB in the immune effects of rocaglates, therefore, remains to be determined.

Targeting elF4 RNA helicase has consequences on T-cell activation. Zotatifin reduced the expression of CD154 (CD40L), the interaction partner of CD40 expressed on antigen-presenting cells, whereas CR-31-B (−) and silvestrol did not affect CD154 expression. This is in line with the published data, as Biswas et al. also found that silvestrol did not regulate CD154 at the mRNA level in activated T cells [32]. Moreover, the pharmacological inhibition of elF4A reduced cytokine release (IL10, IFNγ and IL17A). In T cells, rocaglates inhibit the activation of the transcription factor nuclear factor of activated T cells (NF-AT), which is responsible for the induction of many cytokines (e.g., IL4, IL10, TNFα and IFNγ) and adhesion molecules [33]. Activation of NF-AT proceeds via IP_3_, calcium release and activation of calcineurin, which dephosphorylates NF-AT at serine residues, initiating its translocation to the nucleus [34]. Since NF-AT-dependent cytokines were downregulated in T cells, it is possible that the rocaglates investigated here also interfere with the NF-AT signaling pathway, but we could not detect IP_3_ or Ca^2+^ release in the presence of silvestrol in HEK293T cells [35]. Proksch et al., also failed to observe Ca^2+^ induction in Jurkat T cells with rocaglates [33]. They were able to show that the rocaglates activate JNK/p38 kinases which phosphorylate and thus inactivate NF-AT and that this in turn, prevents the translocation of NF-AT into the nucleus and thus the expression of inflammatory proteins.

An alternative proposal is that NF-AT is activated in a Ca^2+^-independent manner by cytokines such as IL7 [36]. Some rocaglates also inhibit NF-kB activation in Jurkat T cells at higher concentrations (from 100 nM) [30]. Thus, the mechanism by which rocaglates interact with cytokine signaling is still unclear. It is possible that rocaglates reduce cytokine release via interacting with NF-AT directly or via the inhibition of the NF-kB signaling. Moreover, the question of whether the inhibition of T-cell activation is a target- or substance-specific effect cannot be answered conclusively. Although some rocaglates (CR-31-B (−), zotatifin) in our experiments showed similar effects, other rocaglate derivatives such as silvestrol tend to have opposite effects on T cells.

## 4. Materials and Methods

Human primary monocytes, MdMs, MdDCs and dendritic cells (DCs) were cultured in RPMI 1640-Glutamax medium supplemented with 1% penicillin/streptomycin and 10% FCS at 37 °C in a 5% CO_2_ atmosphere. Human primary T cells were cultured in RPMI 1640 medium supplemented with 2 mM of glutamine, 1 % penicillin/streptomycin and 10% FCS at 37 °C in a 5% CO_2_ atmosphere. Human primary B cells were cultured in RPMI 1640 medium supplemented with 2 mM of glutamine, 0.05 mM of ß-mercaptoethanol, 1% penicillin/streptomycin, and 10% FCS at 37 °C in a 5% CO_2_ atmosphere. RPMI 1640-Glutamax, RPMI 1640 medium and glutamine were obtained from Gibco (Thermo Fisher Scientific, Oberhausen, Germany). Buffy coats from healthy donors were obtained fresh from DRK-Blutspendedienst (Frankfurt am Main, Germany). Orangu™ assay was purchased from Cell guidance systems (Cambridge, UK). Human FcR Blocking Reagent, human CD14 MicroBeads, human CD4 MicroBeads, human blood dendritic cell isolation kit II, human B cell isolation kit II, human granulocyte macrophage colony-stimulating factor (GM-CSF), macrophage colony-stimulating factor (M-CSF), bovine serum albumin (BSA), IL4 and all antibodies (except CD54, CD197) for surface staining were purchased from Miltenyi Biotec (Bergisch Gladbach, Germany). Anti-CD54, anti-CD197, IL21 and CCL17 ELISA were obtained from BioLegend (Fell, Germany). A cytometric bead array was purchased from BD Biosciences (Heidelberg, Germany). ELISA for CCL18 was purchased from BosterBio (Pleasanton, CA, USA). PGE_2_ ELISA was purchased from Enzo Life Sciences (Lörrach, Germany). Accutase^®^ solution was purchased from Merck (Darmstadt, Germany). BioColl Trennlösung^®^ was purchased from Bio&Sell (Feucht, Germany). EDTA was purchased from Sigma-Aldrich (Schnellendorf, Germany). sDC40L was obtained from Peprotech (Hamburg, Germany). CpG ODN 2395 was obtained from InvivoGen (Toulouse, France). Rapamycin and unconjugated goat anti-human IgM F(ab’)2 fragments were purchased from Biomol GmbH (Hamburg, Germany). IgG ELISA, IgA ELISA, IL-23 ELISA and CellTrace^TM^ violet were purchased from Thermo Fisher Scientific (Oberhausen, Germany). Silvestrol (purchased from the Sarawak Biodiversity Centre, Kuching, Borneo, at a purity of >99%) was dissolved in DMSO and further diluted in medium (c_stock_ = 6 mM, maximal DMSO concentration during experiments 0.000083 % *v*/*v*). CR-31-B (−) and CR-31-B (+) (a gift from H-G Wendel, MSKCC, NYC, USA) were dissolved in DMSO at a concentration of 10 mM and stored at −20 °C [37].

### 4.1. Isolation of Human CD14^+^ Cells, CD4^+^ Cells, B Cells and Dendritic Cells

Human peripheral blood mononuclear cells (PBMCs) were isolated from fresh buffy coats by density gradient centrifugation as previously described [24]. CD14^+^ and CD4^+^ cells were isolated with human CD14 or CD4 MicroBeads from Miltenyi Biotec (Bergisch Gladbach, Germany) according to the manufacturer’s protocol. Briefly, defined amounts of cells were dissolved in 0.5% BSA/2 mM of EDTA/PBS and incubated with 25 % (*v*/*v*) human CD14 or CD4 MicroBeads for 15 min at 4 °C. After incubation, the magnetic-labeled cells were separated from unlabeled cells via magnetic cell separation (MACS) with LS columns.

B cells and plasmacytoid and myeloid dendritic cells (pDCs and mDCs) were isolated with the B-cell or dendritic-cell isolation kit II (Miltenyi Biotec, Bergisch Gladbach, Germany) according to the manufacturer’s protocol. Briefly, defined amounts of cells were dissolved in 0.5% BSA/2 mM of EDTA/PBS and incubated with 25% (*v*/*v*) non-DC-depletion cocktail (for DCs) for 15 min at 4 °C or with biotin antibody cocktail (for B cells) for 5 min at 4 °C. For B cells, the anti-biotin MicroBeads were added and incubated for 10 min at 4 °C. After incubation, the magnetic-labeled cells were separated from unlabeled cells via magnetic cell separation (MACS) with LS columns. The unlabeled cells (enriched B cells or enriched DCs) were collected. The cell counts were determined using a MACSQuant^®^ Analyzer 10.

### 4.2. Differentiation of Human Macrophages and Dendritic Cells

The differentiation of human monocytes to macrophages (MdMs) or dendritic cells (MdDCs) was performed as previously described [24]. Briefly, for differentiation of MdMs or MdDCs 0.5 × 10^6^ or 0.9 × 10^6^ CD14^+^ cells/well, respectively, were cultivated in the presence of different concentrations of zotatifin, CR-31-B (−), CR-31-B (+) or vehicle (DMSO) in 48-well plates. After preincubation with test substances or DMSO for 30 min, stimulants for differentiation were added as follows: 10 ng/mL of GM-CSF for MdMs or 10 ng/mL of GM-CSF and 10 ng/mL of IL4 for MdDCs and incubation for 7 or 5 days, respectively.

### 4.3. Polarization of Human Macrophages and Activation of MdDCs

The polarization of MdMs to M1 or M2 MdMs and activation of MdDCs were performed as previously described [24]. Briefly, MdMs for M1 polarization were differentiated with 10 ng/mL of human GM-CSF for 7 days from monocytes, MdMs for M2 polarization were differentiated with 50 ng/mL human M-CSF for 7 days from monocytes and MdDCs were differentiated with 10 ng/mL GM-CSF and 10 ng/mL IL4 for 5 days from monocytes. M1 MdMs were polarized in the presence of zotatifin, CR-31-B (−), CR-31-B (+) or vehicle (DMSO) with 20 ng/mL of human IFNγ for 48 h. M2 MdMs were polarized in the presence of zotatifin, CR-31-B (−), CR-31-B (+) or vehicle (DMSO) with 10 ng/mL of human IL4 for 24 h. MdDCs were activated in the presence of zotatifin, CR-31-B (−), CR-31-B (+) or vehicle (DMSO) with a cytokine mixture (10 ng/mL of TNFα, 10 ng/mL of IL1β, 10 ng/mL of IL6, 1 µg/mL of PGE_2_) for 24 h.

### 4.4. Activation of T Cells and B Cells

For T-cell activation, 1.75 × 10^5^ T cells (stained with 0.2 µM CellTrace^TM^ violet (CTV)) per well were pretreated with zotatifin, CR-31-B (−), CR-31-B (+) or vehicle (DMSO) for 30 min in RPMI 1640 medium supplemented with 2 mM of glutamine, 50 units/mL of IL2, 1% penicillin/streptomycin and 10% FCS. Afterward, they were seeded in anti-CD3 (BioLegend, Fell, Germany) coated 96-well plates and stimulated with 2 µg/mL of anti-CD28 (Miltenyi Biotec, Bergisch Gladbach, Germany) for 5 days. CTV staining was performed according to the protocol of the supplier.

B cells were activated as described by Khoenkhoen et al. [38]. First, 1.25 × 10^5^ of B cells (stained with 0.25 µM of CTV) per well were seeded in a 48-well plate and pretreated with zotatifin, CR-31-B (−), CR-31-B (+) or vehicle (DMSO) for 30 min. Then, the B cells were stimulated with 5 μg/mL of unconjugated goat anti-human IgM F(ab’)2 fragments, 2.5 μg/mL of CpG, 1 μg/mL of sCD40L and 50 ng/mL of recombinant human IL21 for 5 days.

### 4.5. Surface Marker Detection and Proliferation Assay with Flow Cytometry

For analysis of the surface markers, 1.5−2 × 10^5^ of cells for each sample was blocked with human FcR Blocking Reagent (15 min, 4 °C). To discriminate living and dead cells, a Zombie Aqua™ Fixable Viability Kit (1:500 dilution, BioLegend, San Diego, CA, USA) or Annexin/PE staining (BD Biosciences, Heidelberg, Germany) was used according to the manufacturer’s protocol. Following this, samples were stained with up to 7 µL of a mixture of different surface marker antibodies (15 min, 4 °C), and 250 µL of 10% FCS/PBS were added. After centrifugation (300 g, 5 min, 4 °C), samples were resuspended in 100 µL of PBS and measured with a MACSQuant^®^ Analyzer 10 flow cytometer. For MdMs, M1-/M2-MdMs anti-CD86, anti-CD14, anti-HLA-DR, anti-CD206, anti-CD80, anti-TREM2 and anti-CD163 were used. For MdDCs and activated DCs, anti-Cd1a, anti-Cd1c, anti-CD54, anti-HLA-DR, anti-CD40, anti-CD83, anti-CD206, anti-CD80, anti-CD209, anti-CD141, anti-CD86 and anti-CD197 were tested. For T cells, anti-CD69, anti-CD45RO, anti-CD134, anti-CD154, anti-CD3 and anti-CD25 were analyzed. For B cells, anti-CD27, anti-CD19 and anti-CD38 were applied. For DCs, anti-CD83, anti-CD80, anti-CD40, anti-CD86 and anti-HLA-DR were tested. For the T-cell and B-cell proliferation assays, freshly isolated T and B cells were stained with CTV and detected with flow cytometry. Naïve B cells were characterized as CD19^+^CD27^low^CD38^med^_,_ memory B cells as CD19^+^CD27^med^ CD38^low^ and plasma cells as CD27^+^CD38^+^ cells. For MdDCs, MdMs, activated DCs, M1 MdMs and M2 MdMs, the cell population was gated using the FSC/SSC channel, while for T cells, CD3^+^CD25^+^ cells were gated. The geometric mean of the fluorescent intensity was calculated using FlowJo software v. 10 (Treestar, Ashland, TN, USA). Fold induction of surface marker expression was calculated using the DMSO-treated cells as a control.

### 4.6. Cytokine, Ig and PGE_2_ Detection with Cytometric Bead Array or ELISA

For determination of cytokine concentrations in the supernatant of differentiated/polarized/activated humane immune cells, cytometric bead array (BD Biosciences, Heidelberg, Germany) for IL10, IL17, IL8, IL6, IL1β, TNFα, IFNγ and CCL2 was used. For the detection of IgG, IgA, PGE_2_, CCL18, CCL17 and IL23, ELISA was performed. The cytometric bead arrays and the ELISAs were performed according to the manufacturer’s protocol.

### 4.7. Determination of Energy Metabolism

Energy metabolism was determined as described previously [24]. Briefly, for the analysis of the extracellular acidification rate (ECAR) and the oxygen consumption rate (OCR) for human M1 MdMs, M2 MdMs and activated MdDCs, the Seahorse XFe96 FluxPak (Agilent, Waldbronn, Germany) was used as recommended by the manufacturer. MdMs were polarized to M1 or M2 MdMs, and DCs were activated in the presence of zotatifin, CR-31-B (−), CR-31-B (+), silvestrol or vehicle (DMSO) for 48 h or 24 h, respectively. Cells were washed with Seahorse XF RPMI medium at a pH of 7.4 (Agilent, Waldbronn, Germany), and OCR and ECAR were measured as octuplicates for a period of 160 min using the Seahorse XFe96 Analyzer (Agilent, Waldbronn, Germany) and were analyzed with Wave 2.6 Software (Agilent, Waldbronn, Germany).

### 4.8. Statistics

Results are presented as means ± standard errors (SEMs). For all calculations and creation of graphs, GraphPad Prism 8 was used, with *p* < 0.05 being considered as the threshold for significance. Applied statistical analyses are denoted in the figure legends. In every test, rocaglate treatment was compared to vehicle.

## 5. Conclusions

Our data clearly demonstrate that the inhibition of the elF4A mRNA helicase with rocaglates influences the function of immune cells, indicating that they can modulate the immune response. In particular, M1 MdM and T-cell and B-cell activation seems to be reduced. In conclusion, in any full preclinical characterization of antiviral drugs, their interactions with the immune system should be addressed carefully. The downregulating effects, which tended to dominate with rocaglates, suggest that these drugs, while inhibiting viral proliferation, may also suppress bystander tissue injury by the host immune system. On the other hand, dosing in vivo would need to be adjusted carefully to prevent excessive immune suppression. In this respect, silvestrol may have some advantages in not exhibiting pronounced immunosuppression.

## Figures and Tables

**Table 1:** Effects of rocaglates on monocyte-derived macrophages.

MdMs	CD86	CD14	HLA-DR	CD206	CD80	TREM2	CD163	IL6	IL10	CCL17	CCL18
CR-31-B (+)	NO	NO	NO	≥0.25	NO	NO	NO	NO	NO	NO	NO
CR-31-B (−)	≥2.5	≥0.5	≥0.5	NO	NO	NO	≥2.5	≥0.25	≥0.25	≥2.5	≥0.5
Silvestrol	NO	* ≥2.5	NO	≥2.5	NO	≥5	≥5	≥0.5	≥0.5	≥0.5	≥0.5
Zotatifin	NO	≥2.5	NO	≥5	≥5	NO	NO	≥5	≥2.5	≥0.5	≥2.5

Silvestrol data are from Blum et al. [24] or unpublished data with 2.5 and 5 nM of silvestrol (indicated with *). ●—reduction; ●—increase; ●—no effect.

**Table 2:** Effects of rocaglates on M1 monocyte-derived macrophages.

M1 MdMs	CD86	CD14	HLA-DR	CD206	CD80	TREM2	CD163	IL6	IFNγ	CCL17	CCL18	CCL2	IL8	TNFα	PGE_2_	OCR	ECAR
CR-31-B (+)	NO	NO	NO	NO	NO	NO	NO	NO	NO	≥2.5	NO	NO	NO	NO	NO	NO	NO
CR-31-B (−)	≥2.5	≥0.5	NO	≥2.5	NO	NO	≥0.5	≥0.5	NO	NO	≥0.5	≥0.5	≥0.5	NO	NO	≥5	≥5
Silvestrol	NO	*≥2.5	NO	NO	NO	≥0.5	NO	*≥5	* NO	* NO	* NO	≥0.5	≥0.5	≥5	* NO	≥5	≥5
Zotatifin	NO	≥5	NO	NO	NO	≥0.5	NO	NO	NO	NO	NO	NO	NO	NO	NO		

Silvestrol data are from Blum et al. [24] or unpublished data with 5 nM of silvestrol (indicated with *). ●—reduction; ●—increase; ●—no effect.

**Table 3:** Effects of rocaglates on M2 monocyte-derived macrophages.

M2 MdMs	CD86	CD14	HLA-DR	CD206	CD80	TREM2	CD163	IL6	IL10	CCL17	CCL18	CCL2	IL8	TNFα	PGE_2_	OCR	ECAR
CR-31-B (+)	NO	NO	NO	NO	NO	NO	NO	NO	NO	NO	NO	NO	NO	NO	NO	NO	NO
CR-31-B (−)	NO	NO	NO	≥5	NO	NO	NO	NO	NO	≥0.5	≥2.5	≥0.5	NO	≥0.5	NO	≥5	≥5
Silvestrol	NO	* NO	NO	≥5	NO	≥5	NO	* NO	* NO	* NO	* NO	≥2.5	≥0.5	NO	* NO	NO	NO
Zotatifin	≥5	NO	NO	NO	NO	NO	NO	NO	≥2.5	NO	≥2.5	NO	NO	NO	NO		

Silvestrol data are from Blum et al. [24] or unpublished data with 5 nM of silvestrol (indicated with *). ●—reduction; ●—increase; ●—no effect.

**Table 4:** Effects of rocaglates on the differentiation of monocytes to dendritic cells.

MdDCs	Cd1a	Cd1c	CD54	HLA-DR	CD40	CD83	CD206	CD80	CD209	CD141	CD86	CD197	IL1β	CCL17	CCL18	IL6	IL10	IL8
CR-31-B (+)	NO	≥5	NO	NO	NO	NO	NO	NO	NO	NO	NO	NO	NO	NO	NO	NO	NO	NO
CR-31-B (−)	≥2.5	≥0.5	≥0.5	≥5	≥5	≥5	NO	≥2.5	≥0.5	≥5	≥0.5	NO	≥5	≥2.5	≥0.5	NO	NO	NO
Silvestrol	≥2.5	≥0.5	≥2.5	≥2.5	≥5	≥2.5	≥2.5	NO	≥0.5	≥5	≥2.5	NO	≥2.5	* NO	*≥5	≥2.5	≥2.5	≥2.5
Zotatifin	≥0.5	≥2.5	≥2.5	NO	NO	NO	≥5	NO	≥2.5	≥5	NO	NO	NO	NO	≥2.5	NO	≥0.5	NO

Silvestrol data are from Blum et al. [24] or unpublished data with 5 nM of silvestrol (indicated with *). ●—reduction; ●—increase; ●—no effect.

**Table 5:** Effects of rocaglates on activated monocyte-derived dendritic cells.

Act. MdDCs	Cd1a	Cd1c	CD54	HLA-DR	CD40	CD83	CD206	CD80	CD209	CD141	CD86	CD197	IL1β	CCL17	CCL8	IL6	IL10	IL8	IL23	OCR	ECAR
CR-31-B (+)	NO	NO	NO	NO	NO	NO	NO	NO	NO	NO	NO	NO	NO	NO	NO	NO	NO	NO	NO	NO	NO
CR-31-B (−)	≥2.5	≥2.5	≥2.5	≥5	NO	≥0.5	NO	≥2.5	NO	≥5	NO	NO	NO	≥0.5	NO	≥2.5	≥2.5	≥0.5	≥5	NO	NO
Silvestrol	NO	NO	≥2.5	NO	≥2.5	NO	NO	NO	≥5	NO	≥5	NO	* NO	* NO	* NO	≥2.5	≥5	≥0.5	≥2.5	NO	NO
Zotatifin	≥2.5	NO	NO	NO	NO	NO	NO	NO	NO	NO	NO	NO	NO	NO	NO	NO	NO	NO	NO		

Silvestrol data are from Blum et al. [24] or unpublished data with 5 nM of silvestrol (indicated with *). ●—reduction; ●—increase; ●—no effect.

**Table 6:** Effects of rocaglates on activated T cells.

T Cells	Apoptosis	Proliferation	CD3+ CD25+ Cells	CD45RO	CD69	CD134	CD154	IL17	IL10	IFNγ
CR-31-B (+)	NO	NO	NO	NO	NO	NO	NO	NO	NO	NO
CR-31-B (−)	≥2.5	≥2.5	≥2.5	≥0.5	NO	≥2.5	NO	≥0.5	≥2.5	≥2.5
Silvestrol	NO	NO	NO	≥5	NO	NO	NO	NO	≥5	≥5
Zotatifin	NO	≥5	≥2.5	≥5	≥5	NO	≥5	≥5	≥5	≥5

●—reduction; ●—increase; ●—no effect.

**Table 7:** Effects of rocaglates on activated B cells.

B Cells	Apoptosis	Proliferation	Memory B Cells	Naive B Cells	Plasma Cells	IgG	IgA
CR-31-B (+)	NO	NO	NO	NO	NO	NO	NO
CR-31-B (−)	NO	≥2.5	NO	≥2.5	≥0.5	≥0.5	≥0.5
Silvestrol	NO	≥0.5	NO	NO	≥2.5	≥2.5	≥2.5
Zotatifin	NO	≥0.5	NO	NO	≥2.5	≥0.5	≥0.5

●—reduction; ●—increase; ●—no effect.

**Table 8:** Co-culture of DCs with T cells: Effects on dendritic cells (day 2 and day 7).

DCs	Apoptosis [d2]	CD83 [d2]	CD80 [d2]	CD40 [d2]	HLA-DR [d2]	CD86 [d2]	Apoptosis [d7]	CD83 [d7]	CD80 [d7]	CD40 [d7]	HLA-DR [d7]	CD86 [d7]
CR-31-B (+)	NO	NO	NO	≥5	NO	NO	NO	NO	NO	NO	NO	NO
CR-31-B (−)	NO	≥0.5	NO	≥5	≥5	≥5	≥2.5	≥0.5	≥0.5	≥2.5	≥2.5	NO
Silvestrol	NO	≥2.5	NO	≥5	NO	NO	NO	NO	NO	NO	NO	NO
Zotatifin	NO	≥0.5	NO	NO	NO	NO	NO	≥5	≥2.5	≥2.5	NO	NO

●—reduction; ●—increase; ●—no effect.

**Table 9:** Coculture of DCs with T cells: effects on T cells (day 7).

T Cells	Apopt.	Prolif.	CD3+ CD25+ Cells	CD45RO	CD69	CD 134	CD154	IL17	IL10	IFNγ
CR-31-B (+)	NO	NO	NO	NO	NO	NO	NO	NO	NO	NO
CR-31-B (−)	≥2.5	≥0.5	≥0.5	≥2.5	≥2.5	NO	≥5	≥2.5	≥2.5	≥0.5
Silvestrol	NO	NO	≥5	NO	NO	NO	NO	NO	≥5	NO
Zotatifin	NO	≥2.5	≥2.5	≥2.5	NO	NO	≥5	≥2.5	≥5	≥0.5

●—reduction; ●—increase; ●—no effect.

## Data Availability

The data that support the findings of this study are available from the corresponding author upon reasonable request.

## Supplementary Materials

The following supporting information can be downloaded at https://www.mdpi.com/article/10.3390/ijms24065872/s1.
