# Supplementary material for: Comparing the Effects of Rocaglates on Energy Metabolism and Immune Modulation on Cells of the Human Immune System
Source: Int J Mol Sci. 2023 Mar 20;24(6):5872. doi: 10.3390/ijms24065872 (PMC10051175; doi:10.3390/ijms24065872)
Supplement: Supplementary file 1 [file ijms-24-05872-s001.zip › ijms-2221032-supplementary.pdf]

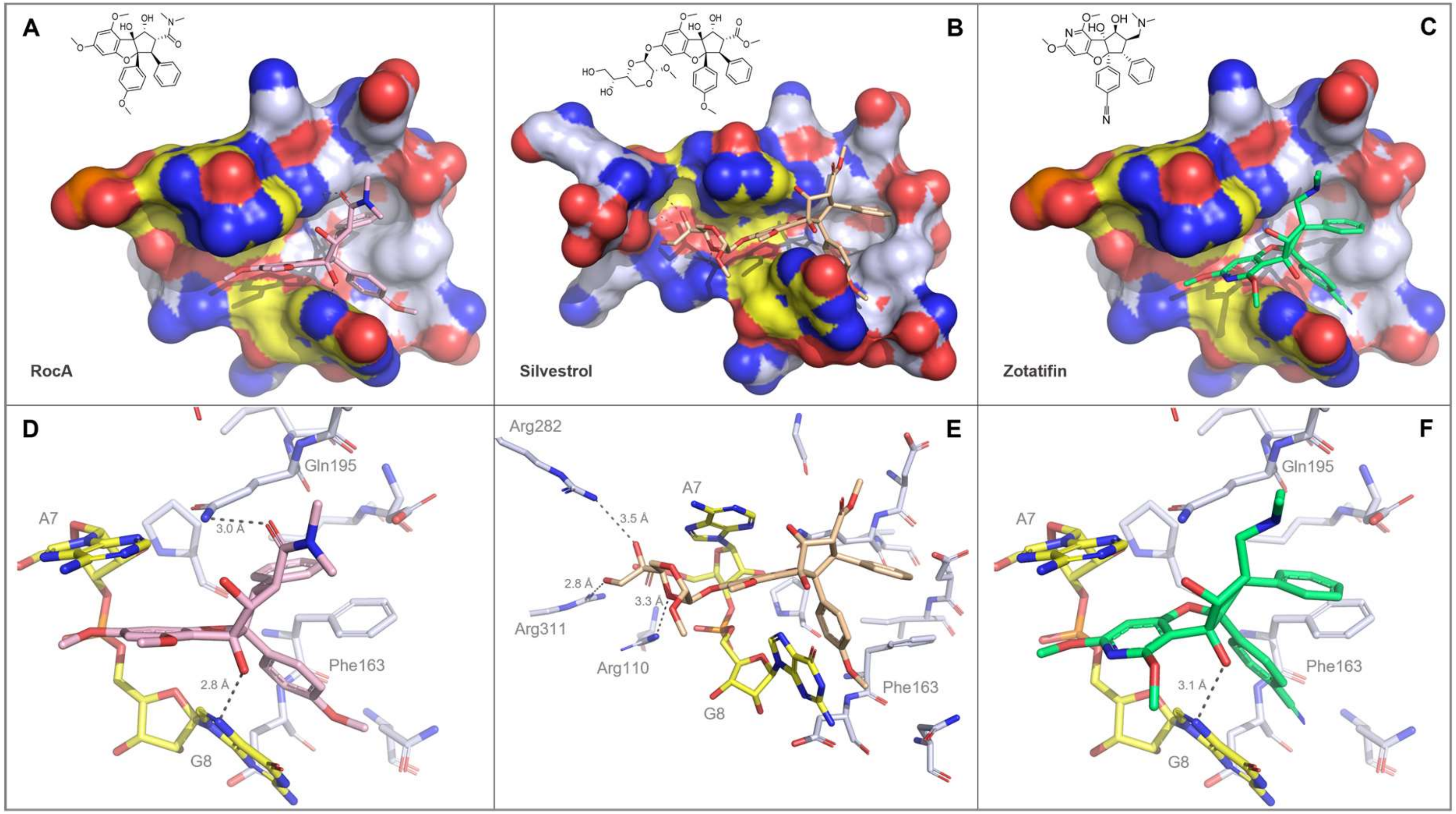

Supplemental Figure S1: Surface (**A, B, C**) and stick (**D, E, F**) representation of the natural rocaglate RocA (pink stick in **A** and **D**) and Silvestrol (wheat stick in **B** and **E**) and the synthetic rocaglate Zotatifin (green stick in **C** and **F**) in the rocaglate binding pocket. Rocaglate are mainly involved in π-π face-to-face

and face-to-edge stacking interactions with Phe163 and with A7 and G8 of the polypurine RNA $(AG)_5$. Phe163 is an essential residue for the rocaglate clamping and binding mechanism on the eIF4A-RNA surface. Silvestrol has an additional dioxane moiety which is presumably involved in further interactions with a highly conserved Arginine pocket on the surface of eIF4A which consists of Arg110, Arg282 and Arg311. Binding mode of Silvestrol and Zotatifin are based on docking while the one of RocA on crystal structure (PDB: 5ZC9).

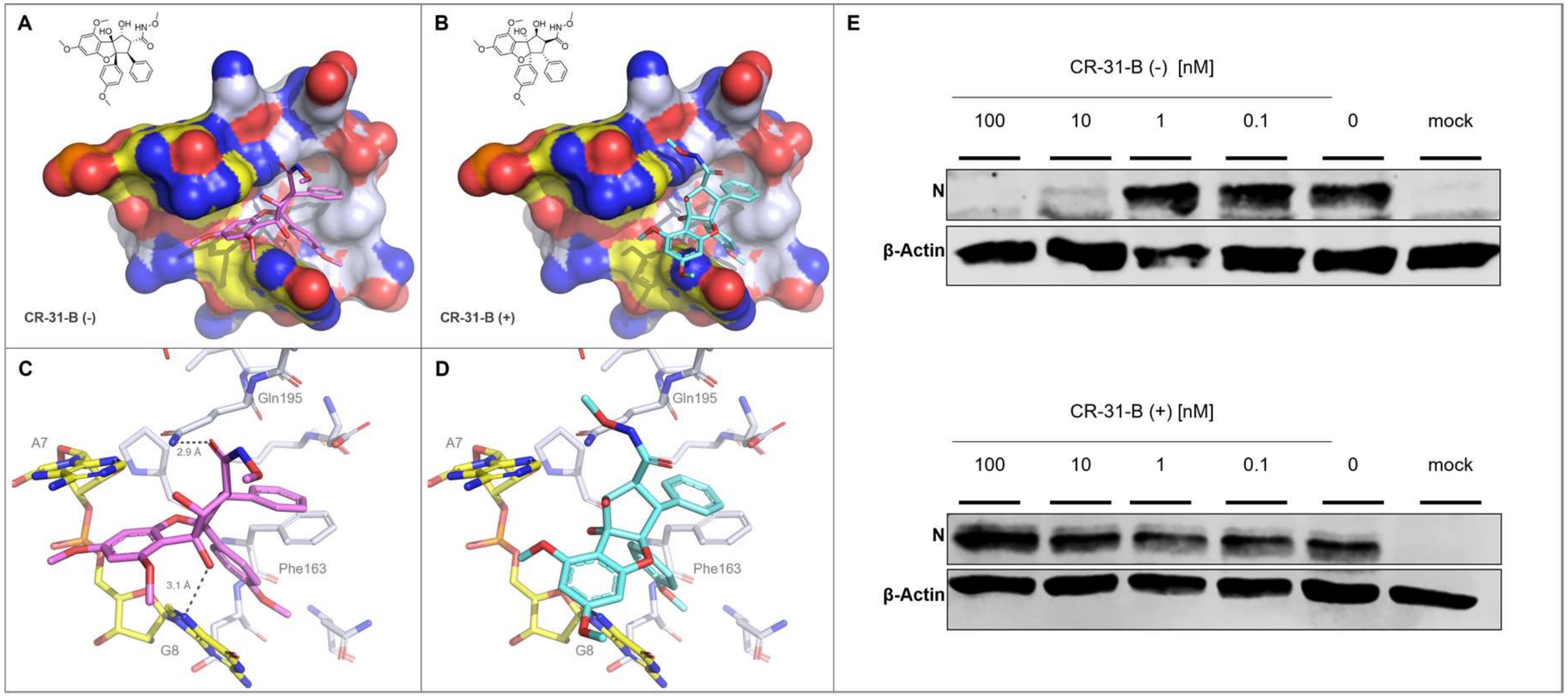

Supplemental Figure S2: Surface (A, B) and stick (C, D) representation of CR-31-B (-) (violet stick in A and C) and CR-31-B (+) (cyan stick in B and D) in the rocaglate binding pocket. T bio o ica y activ (−)-enantiomer (A, D) is involved in two hydrogen bonds (grey dashed lines) with Gln195 and RNA G8. The biologically inactive (+)-enantiomer (B, D) does not interact neither with the protein nor with the RNA. As other rocaglates (Suppl. Fig. 1.), CR-31-B (-) is also involved in π- π stacki i t actio s with Phe163, A7 and G8. The docking pose illustrates the different conformation assumed by the enantiomers in the rocaglate binding pocket, which accounts for their different activity. (E) Comparison of broad-spectrum antiviral activities of the synthetic rocaglate CR-31-B (−) a d CR-31-B (+) using western blot technique (Müller et al., Antiviral Research, 2020).

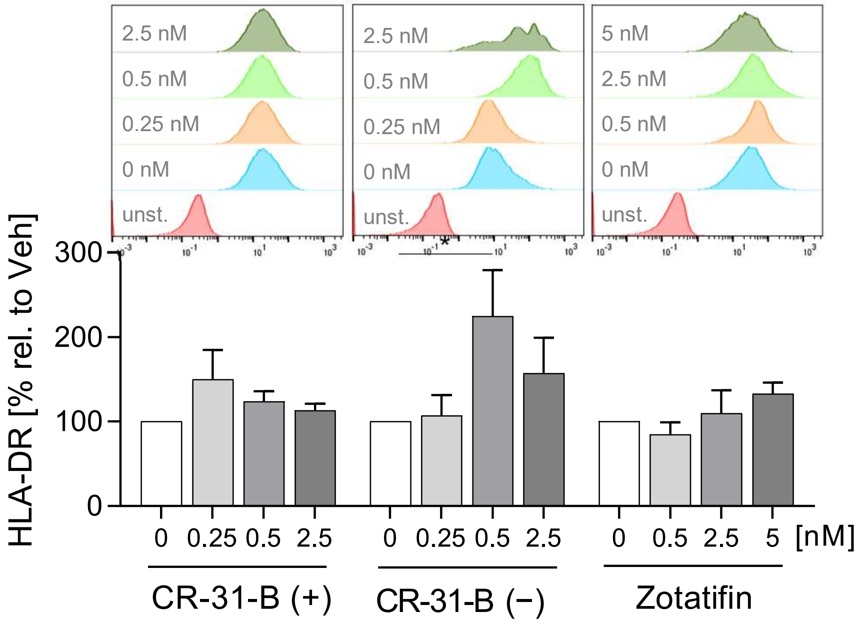

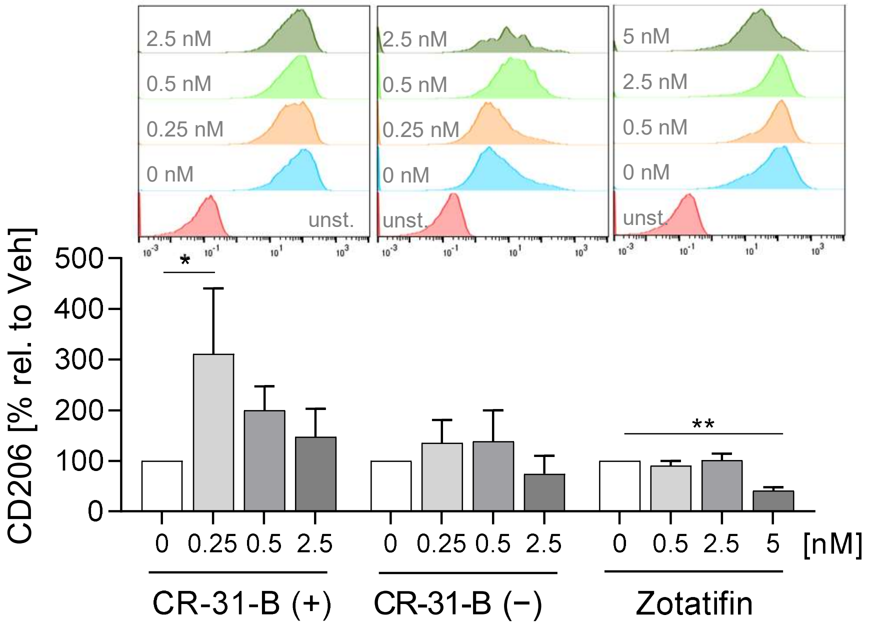

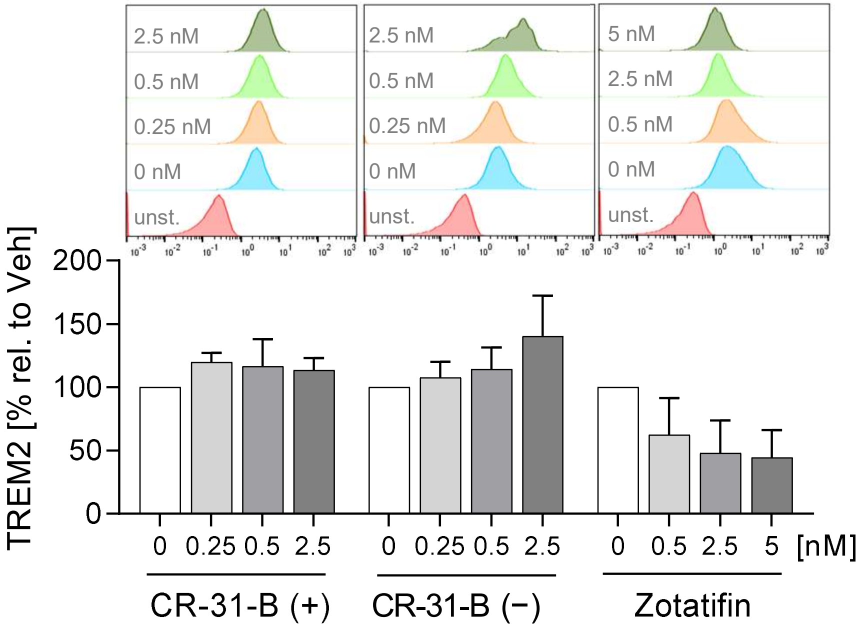

Supplemental Figure S3: Effect of rocaglates on surfaces marker expression on MdMs. Monocytes were isolated from buffy coates and differentiated with 10 ng/ml GM-CSF to MdMs in presence or absence of rocaglates in the indicated concentrations in 7 days . The surface marker expression (MFI) was determined by flow cytometry. The geometric mean of the surface markers of rocaglate treated samples were related to the vehicle control. n=3-5. Data are shown as mean ± SEM. For statistical analysis, mixed-effect analysis with Dunnett's multiple comparisons test was used. * $p<0.05$, ** $p<0.01$, ***$p<0.001$ indicate significant difference between rocaglates and vehicle-treated samples.

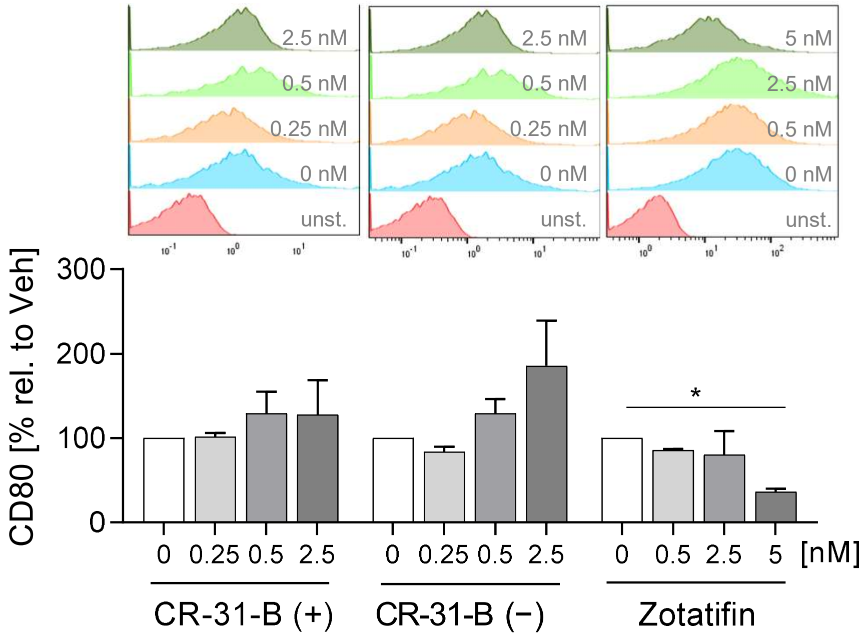

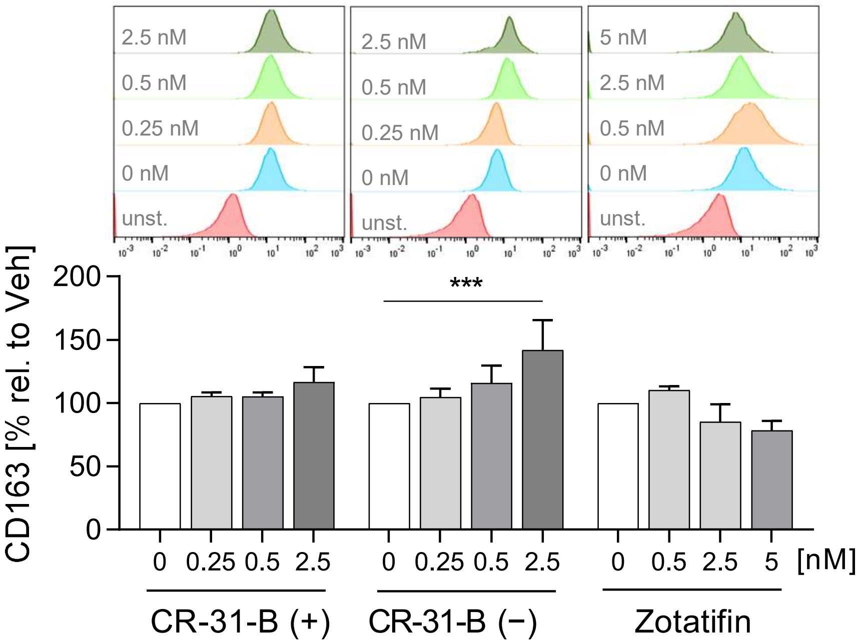

Continuation Supplemental Figure S3

(A)

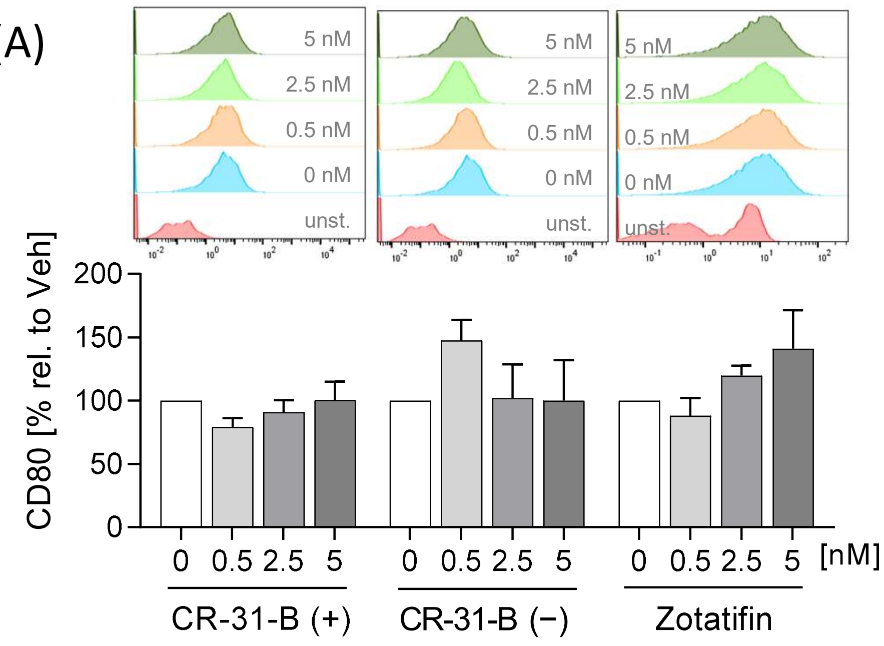

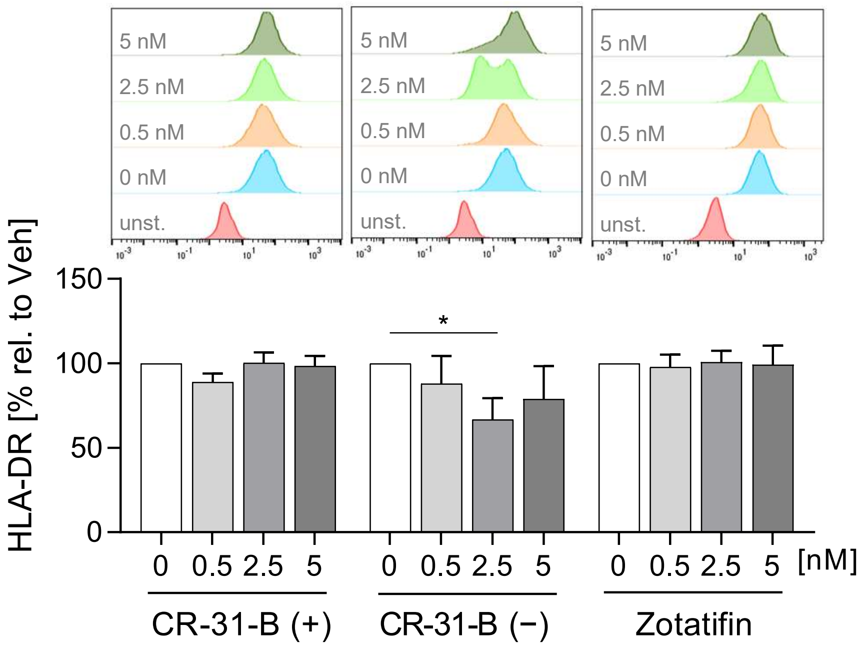

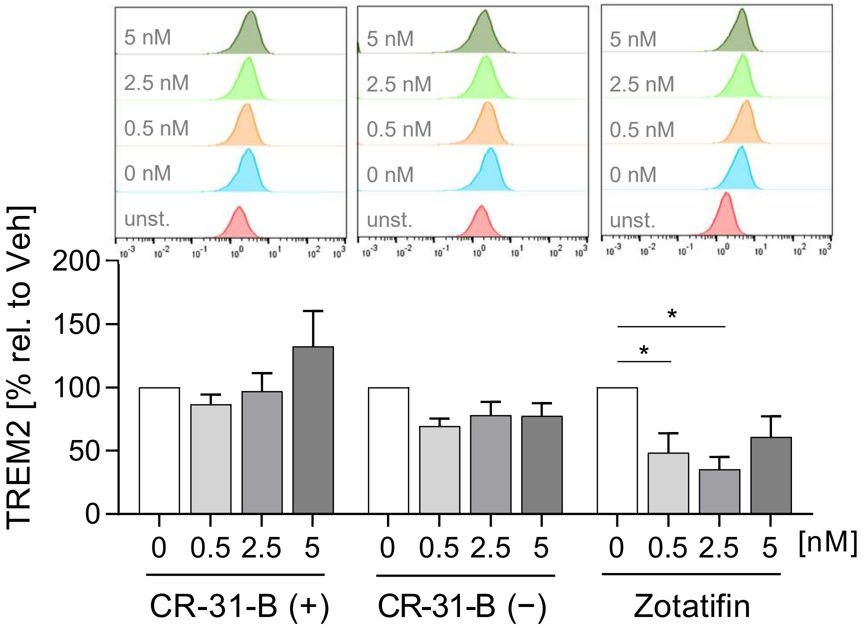

Supplemental Figure S4: Effect of rocaglates on surfaces marker expression and cytokine release in M1 MdM. Monocytes were isolated from buffy coates and differentiated with 50 ng/ml GM-CSF to MdMs for 7 days and activated with 20 ng/ml IFNγ in presence or absence of rocaglates in the indicated concentrations for 2 days . A) The surface marker expression (MFI) was determined by flow cytometry. The geometric mean of the surface markers of rocaglate treated samples were related to the vehicle control. B) The cytokine levels were determined with cytometric bead array. n=3-5. Data are shown as mean ± SEM. For statistical analysis, mixed-effect analysis with Dunnett's multiple comparisons test was used. * $p<0.05$, ** $p<0.01$, *** $p<0.001$ indicate significant difference between rocaglates and vehicle treated samples.

(B)

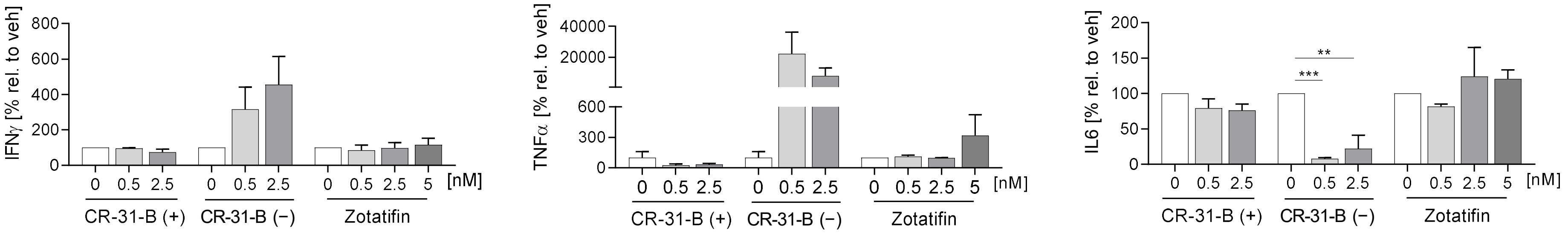

Continuation Supplemental Figure S4

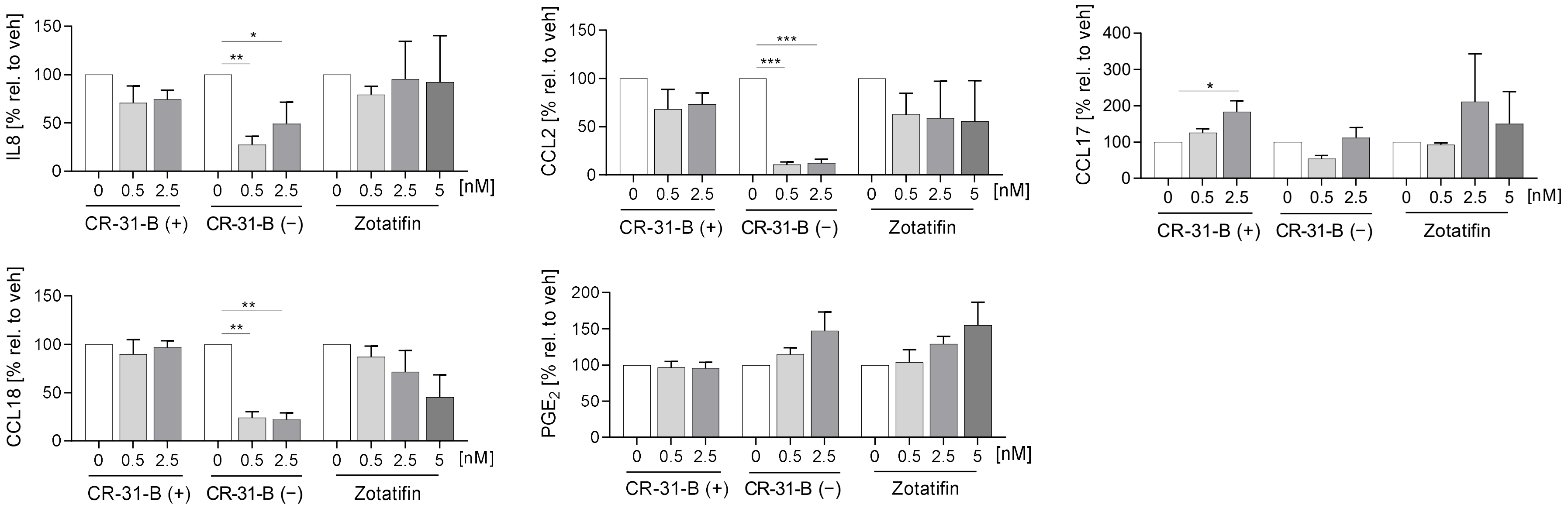

Supplemental Figure S5: Effect of rocaglates on cytokine release and inflammatory mediator of M1 MdMs. Monocytes were isolated from buffy coates and differentiated with 50 ng/ml GM-CSF to MdMs during 7 days and activated with 20 ng/ml IFNγ in presence or absence of rocaglates in the indicated concentrations for 2 days. The cytokines were determined with cytometric bead array. PGE_2 was determined with ELISA. The concentration of rocaglate treated samples were related to the vehicle control. n=3-6. Data are shown as mean ± SEM. For statistical analysis, mixed-effect analysis with Dunnett's multiple comparisons test was used. * $p<0.05$, ** $p<0.01$, ***$p<0.001$ indicate significant difference between rocaglates and vehicle treated samples.

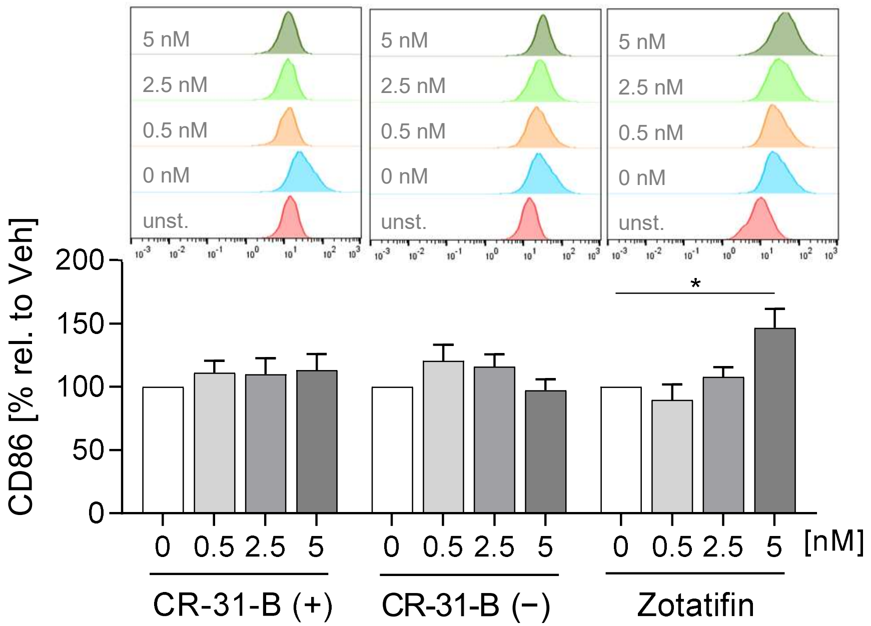

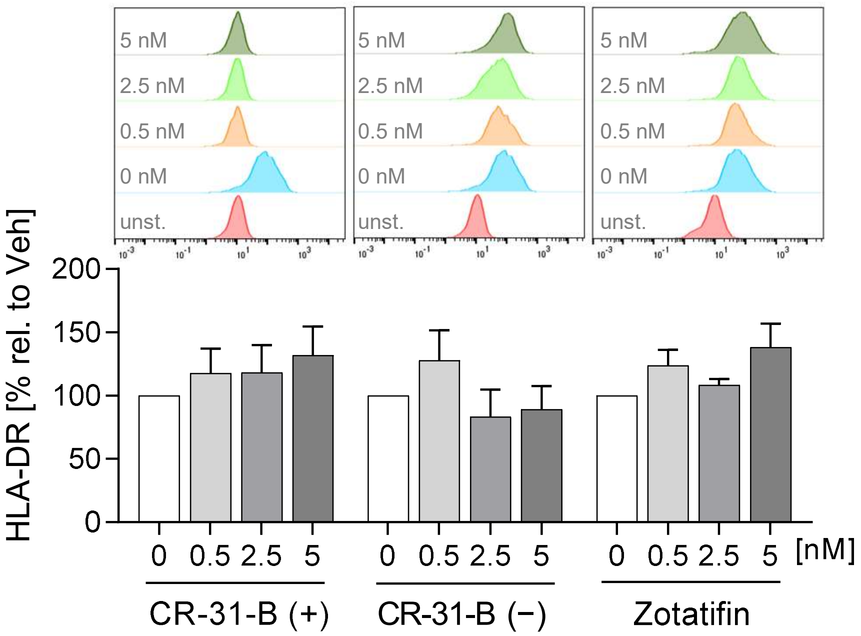

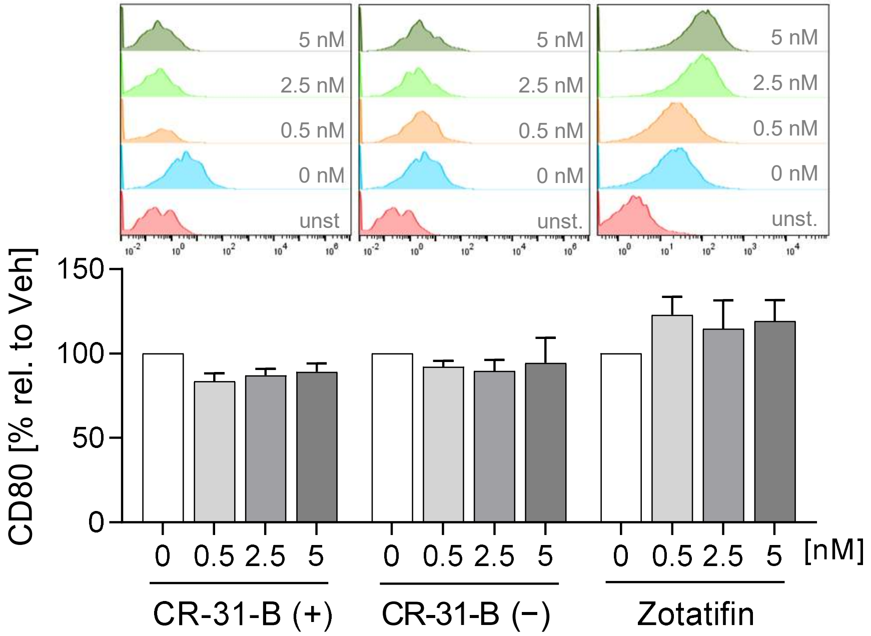

Supplemental Figure S6: Effect of rocaglates on surfaces marker expression on M2 MdM. Monocytes were isolated from buffy coates and differentiated with 50 ng/ml M-CSF to MdMs in 7 days and activated with 10 ng/ml IL4 in presence or absence of rocaglates in the indicated concentrations for 1 day . The surface marker expression (MFI) was determined by flow cytometry. The geometric mean of the surface markers of rocaglate treated samples were related to the vehicle control. n=3-5. Data are shown as mean ± SEM. For statistical analysis, mixed-effect analysis with Dunnett's multiple comparisons test was used. * p<0.05, ** p<0.01, ***p<0.001 indicate significant difference between rocaglates and vehicle treated samples.

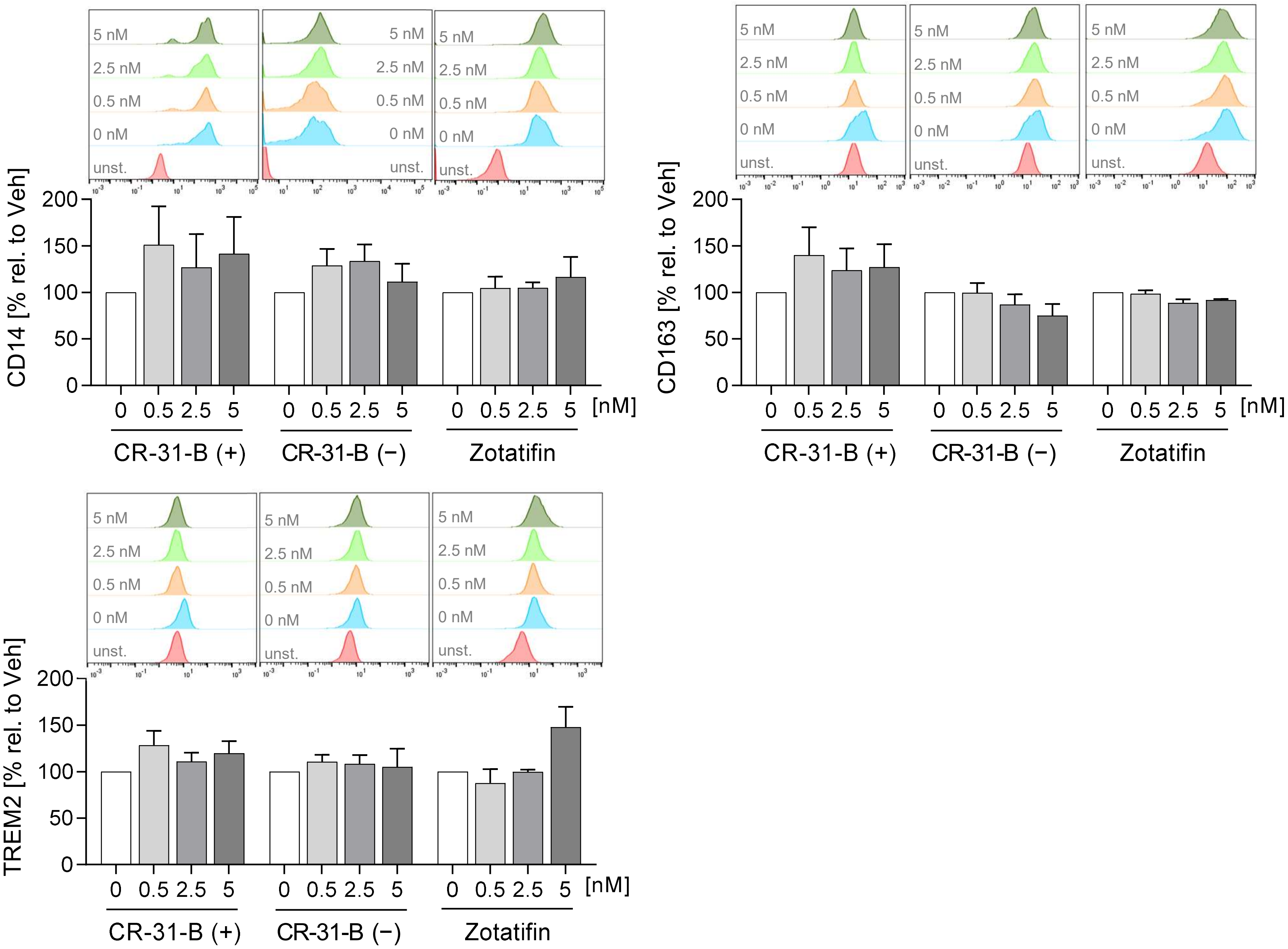

Continuation Supplemental Figure S6

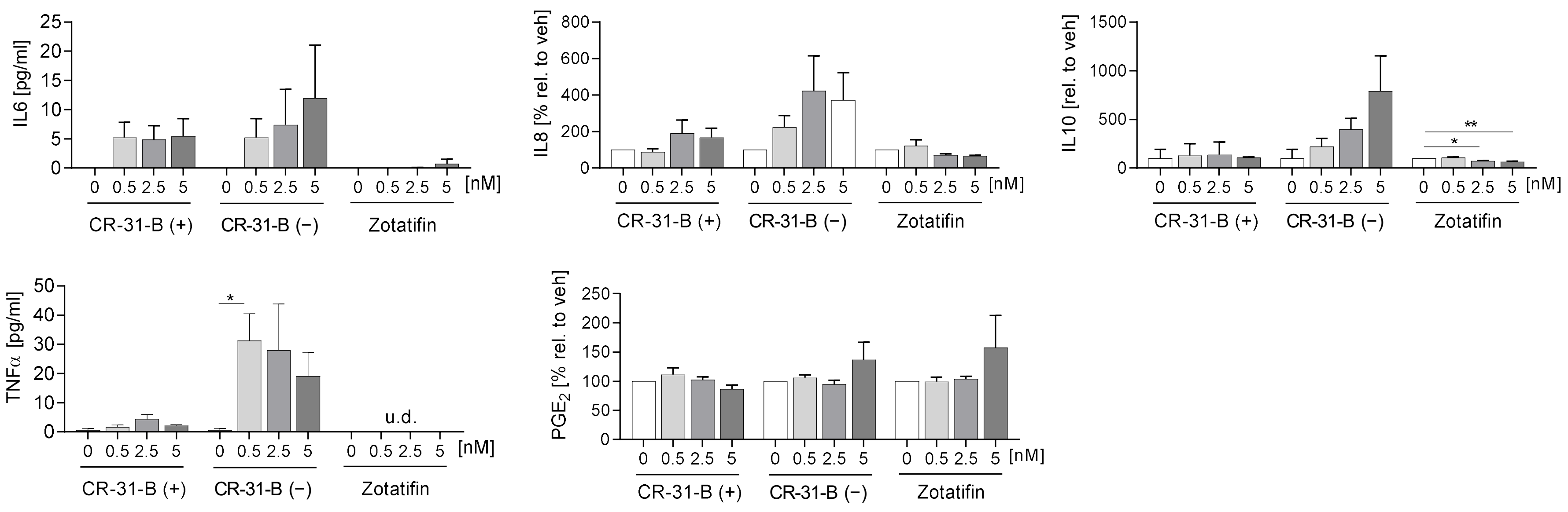

Supplemental Figure S7: Effect of rocaglates on cytokine and inflammatory mediator release of M2 MdMs. Monocytes were isolated from buffy coates and differentiated with 50 ng/ml M-CSF to MdMs in 7 days and activated with 10 ng/ml IL4 in presence or absence of rocaglates in the indicated concentrations for 1

day. The cytokines were determined with cytometric bead array, whereas PGE_2 was determined by ELISA. For IL8, IL10 and PGE_2, the concentrations of rocaglate treated samples were related to the vehicle control. For IL6 and TNFα, the vehicle treated samples were under the detection limit and therefore the pg/ml values are shown instead of % values. n=3-6. Data are shown as mean ± SEM. For statistical analysis, mixed-effect analysis with Dunnett's multiple comparisons test was used. * p<0.05, ** p<0.01, ***p<0.001 indicate significant difference between rocaglates and vehicle treated samples.

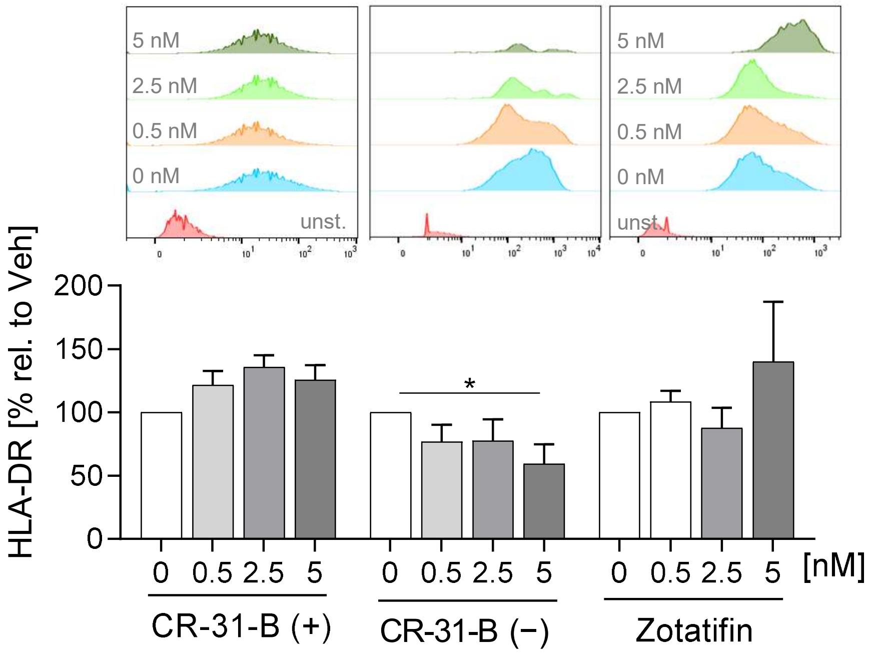

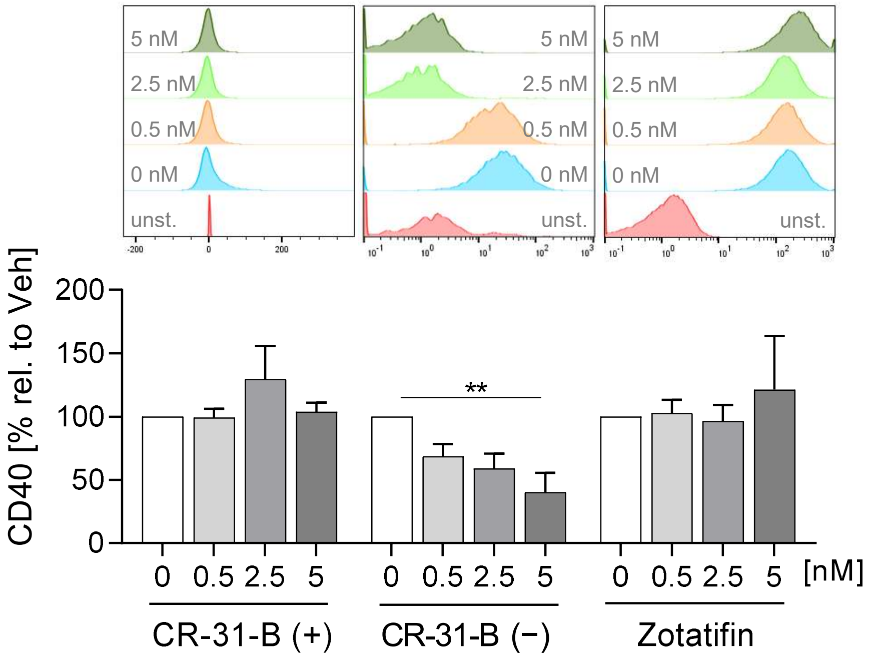

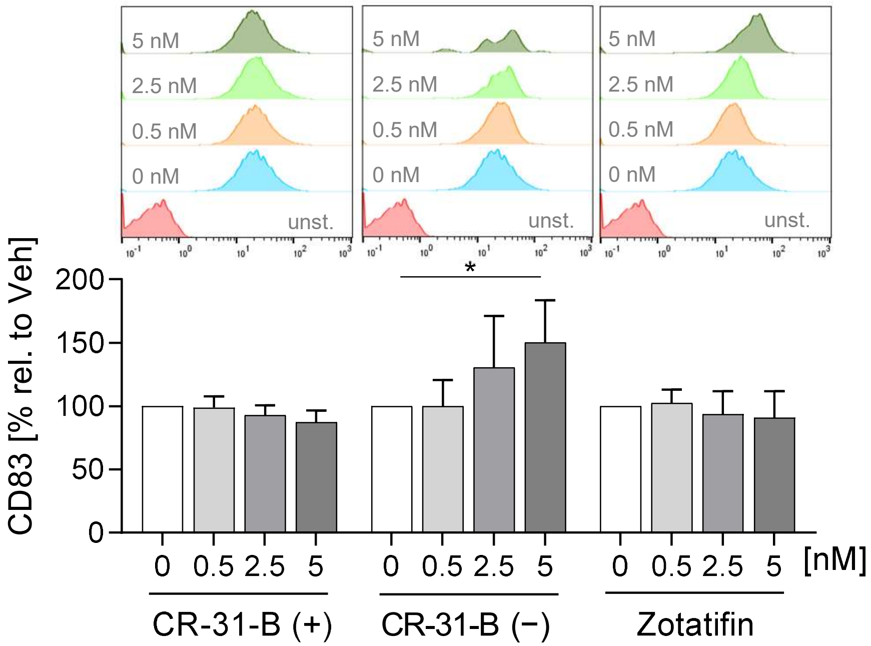

Supplemental Figure S8: Effect of rocaglates on surfaces marker expression on MdDCs. Monocytes were isolated from buffy coats and differentiated with 50 ng/ml GM-CSF and 50 ng/ml IL-4 to MdDCs for 5 days in presence or absence of rocaglates in the indicated concentrations. The surface marker expression (MFI) was determined by flow cytometry. The geometric mean of the surface markers of rocaglate treated samples were related to the vehicle control. n=3-6. Data are shown as mean ± SEM. For statistical analysis, mixed-effect analysis with Dunnett's multiple comparisons test was used. * p<0.05, ** p<0.01 indicate significant difference between rocaglates and vehicle treated samples.

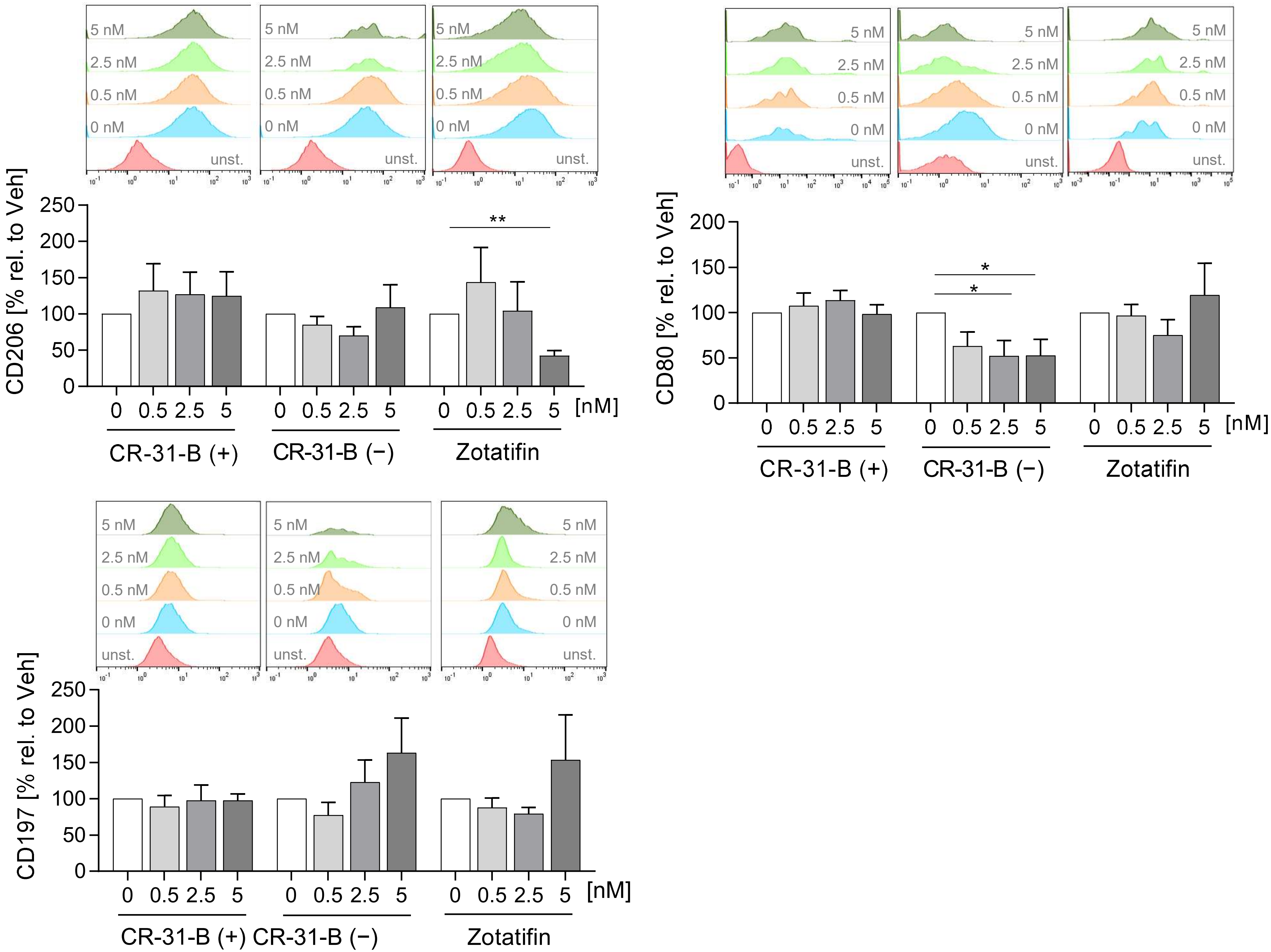

Continuation Supplemental Figure S8

(A)

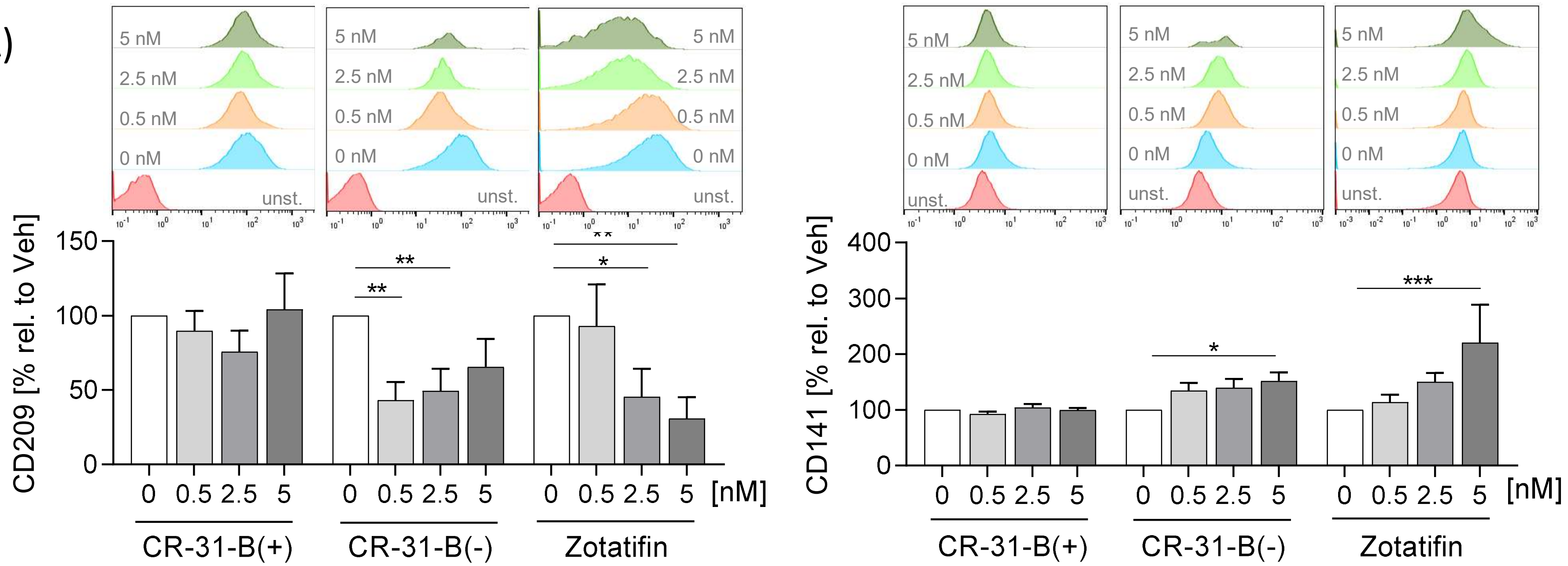

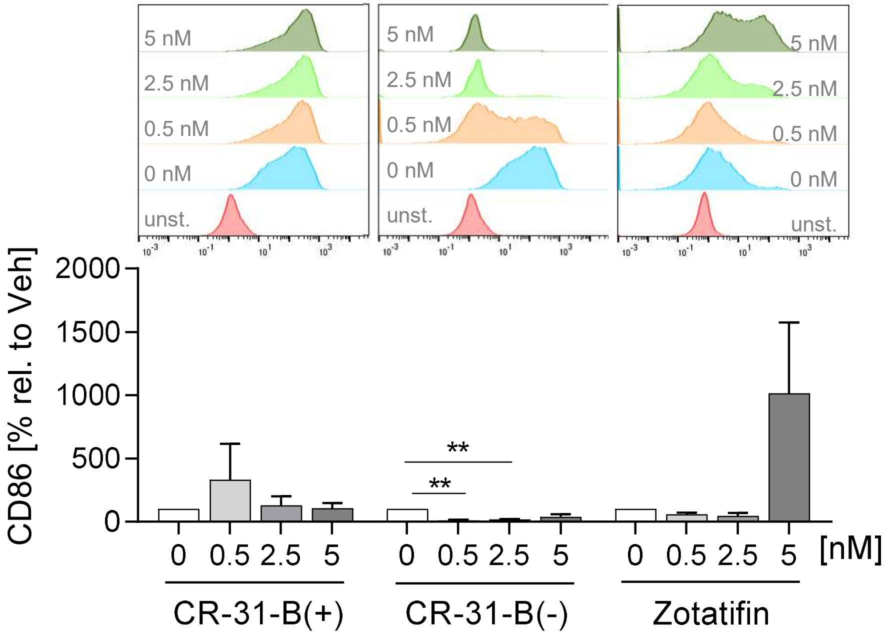

Supplemental Figure S9: Effect of rocaglates on surfaces marker expression (A) and cytokine release (B) on MdDCs. Monocytes were isolated from buffy coates and differentiated with 50 ng/ml GM-CSF and 50 ng/ml IL4 to MdDCs in presence or absence of rocaglates in the indicated concentrations for 5 days. The surface marker expression (MFI) was determined by flow cytometry. The geometric mean of the surface markers of rocaglate treated samples were related to the vehicle control. The cytokines were determined with cytometric bead array. The concentration of rocaglate treated samples were related to the vehicle control. n=3-6. Data are shown as mean ± SEM. For statistical analysis, mixed-effect analysis with Dunnett's multiple comparisons test was used. * p<0.05, ** p<0.01, ***p<0.001 indicate significant difference between rocaglates and vehicle treated samples.

(B)

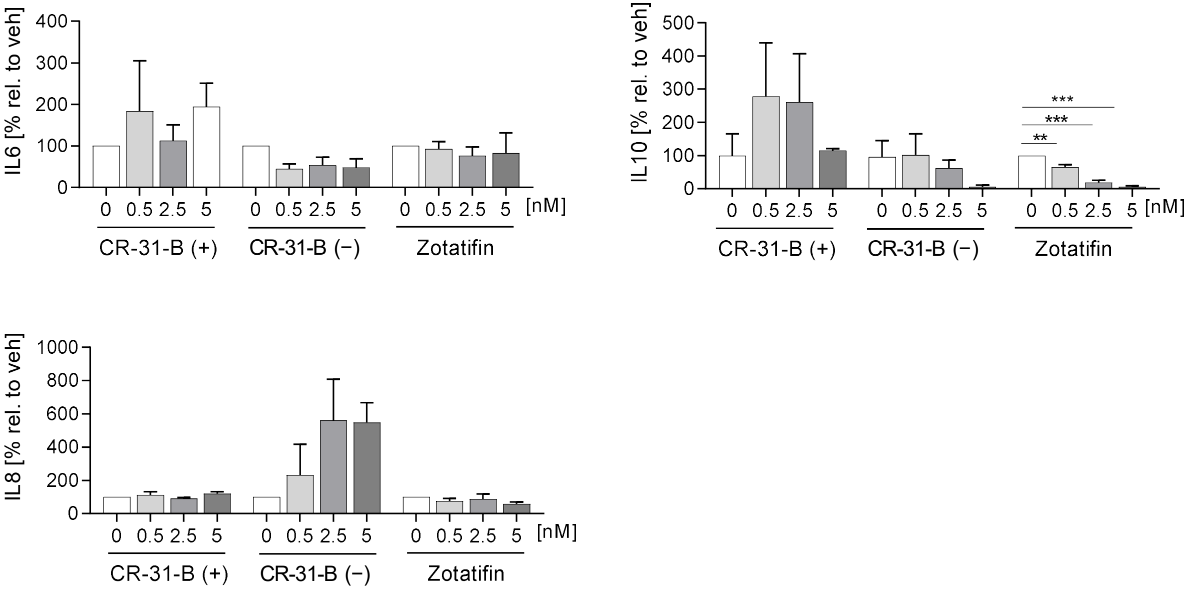

Continuation Supplemental Figure S9

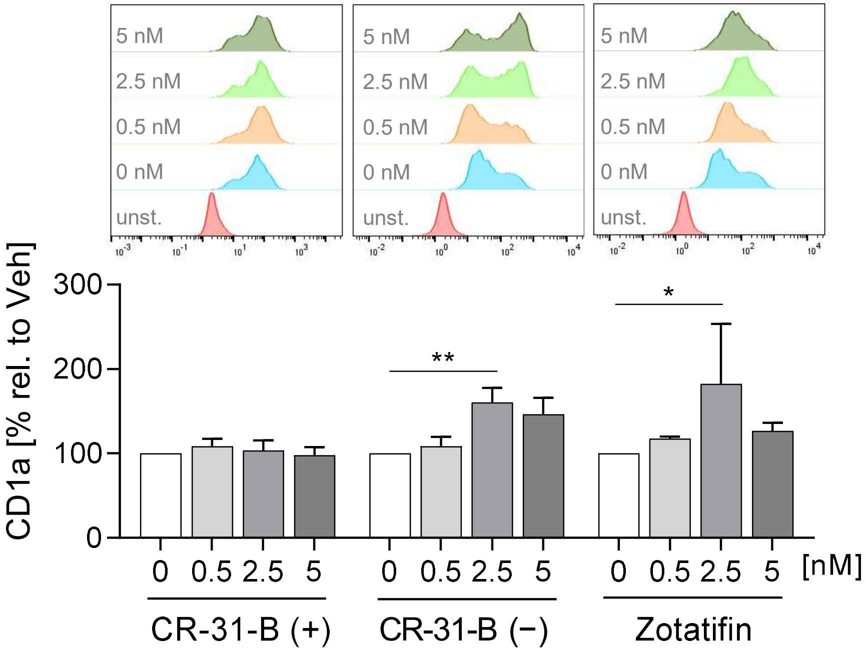

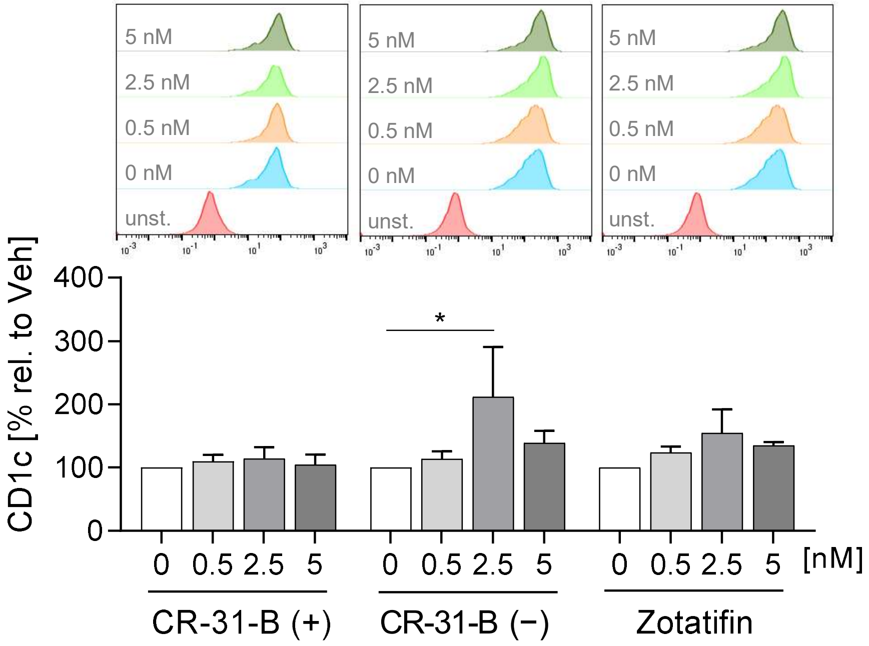

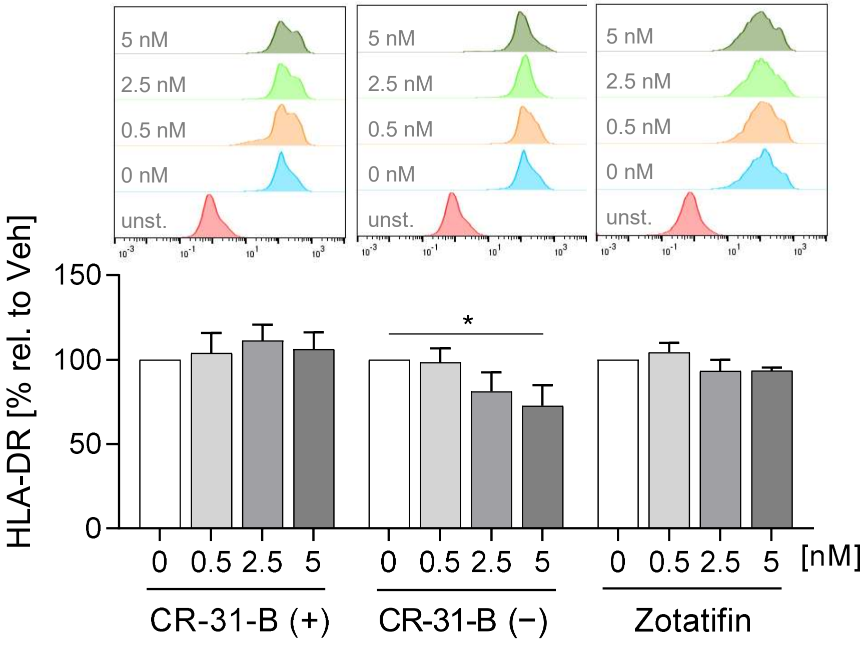

Supplemental Figure S10: Effect of rocaglates on surfaces marker expression on activated MdDCs. Monocytes were isolated from buffy coates and differentiated with 50 ng/ml GM-CSF and 50 ng/ml IL4 to MdDCs in 5 days. MdDCs were activated with a
cytokine mixture (1 µg/ml PGE_2, 10 ng/ml IL1β, 10 ng/ml TNFα, 10 ng/ml IL6) in presence or absence of rocaglates in the indicated concentrations for 1 day . The surface marker expression (MFI) was determined by flow cytometry. The geometric mean of the surface markers of rocaglate treated samples were related to the vehicle control. n=3-6. Data are shown as mean ± SEM. For statistical analysis mixed-effect analysis with Dunnett's multiple comparisons test was used. * p<0.05, ** p<0.01 indicate significant difference between rocaglates and vehicle treated samples.

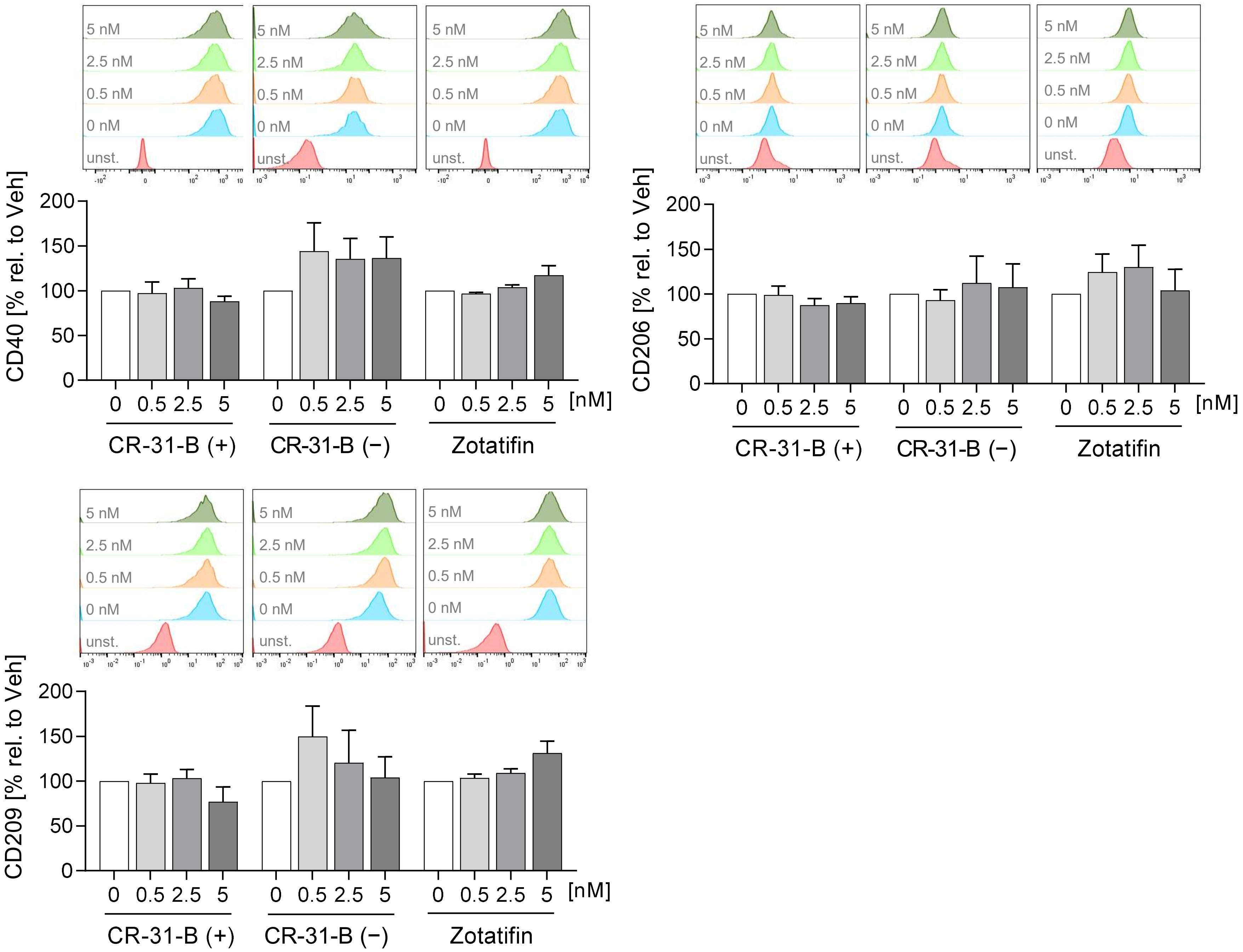

Continuation Supplemental Figure S10

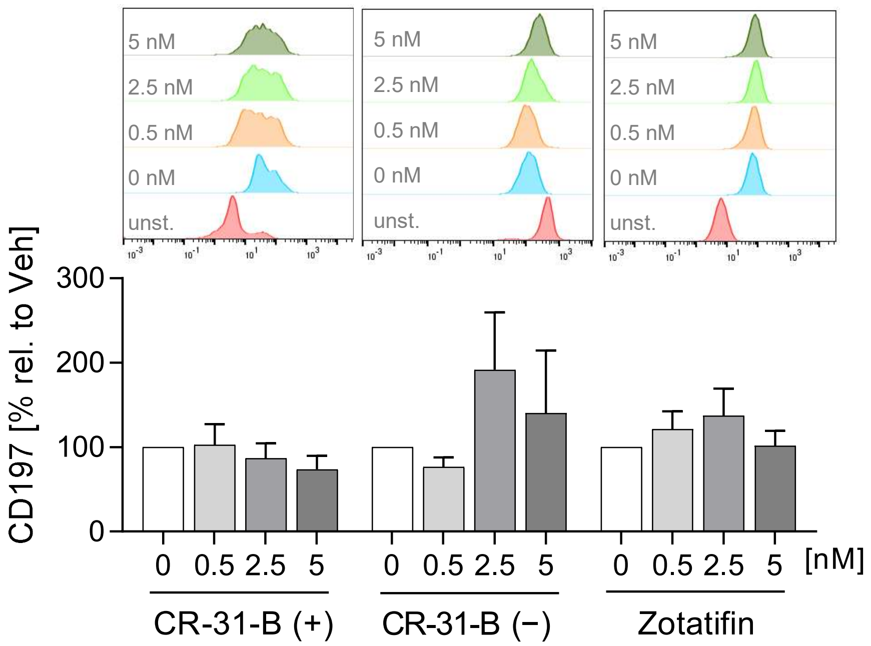

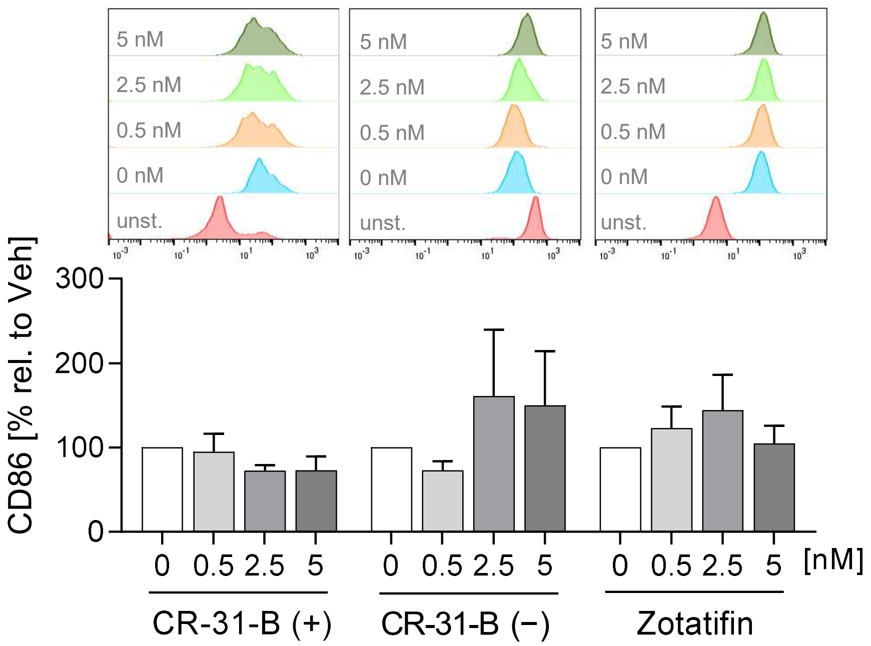

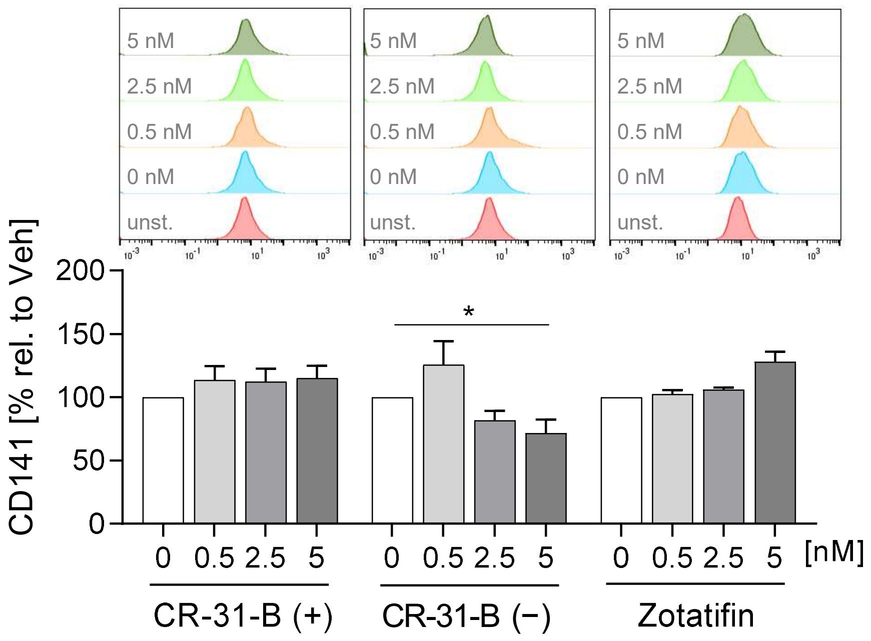

Supplemental Figure S11: Effect of rocaglates on surfaces marker expression on activated MdDCs. Monocytes were isolated from buffy coates and differentiated with 50 ng/ml GM-CSF and 50 ng/ml IL4 to MdDCs in 5 days. Monocytes derived dendritic cells were activated with a cytokine mixture (5
µg/ml PGE_2, 10 ng/ml IL1β, 10 ng/ml TNFα,10 ng/ml IL6) in presence or absence of rocaglates in the indicated concentrations for 1 day. The surface marker expression (MFI) was determined by flow cytometry. The geometric mean of the surface markers of rocaglate treated samples were related to the vehicle control. n=3-6. Data are shown as mean ± SEM. For statistical analysis mixed-effect analysis with Dunnett's multiple comparisons test was used. * p<0.05, ** p<0.01, ***p<0.001 indicate significant difference between rocaglates and vehicle treated samples.

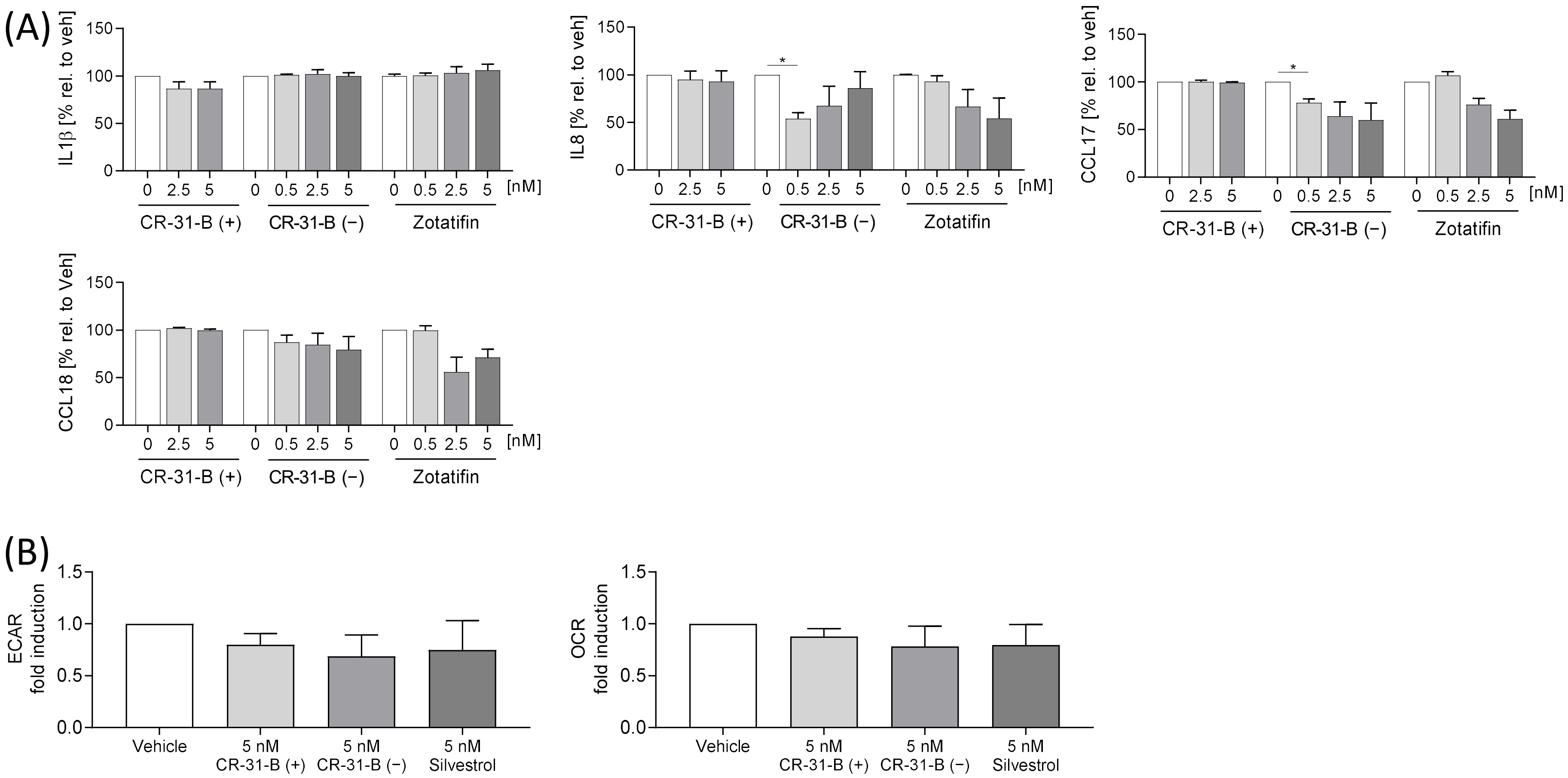

Supplemental Figure S12: Effect of rocaglates on cytokine release and energy metabolism of activated MdDCs. Monocytes were isolated from buffy coates and differentiated with 50 ng/ml GM-CSF and 50 ng/ml IL4 to MdDCs for 5 days. Monocytes derived dendritic cells were activated with a cytokine mixture (5 µg/ml PGE_2, 10 ng/ml IL1β, 10 ng/ml TNFα,10 ng/ml IL6) in presence or absence of rocaglates in the indicated concentrations for 1 day. A) The cytokines were determined with cytometric bead array. B) The oxygen consumption rate (OCR) and extracellular acidification rate (ECAR) were measured with the Seahorse XFe96 analyzer (Agilent, Waldbronn, Germany). The value of the rocaglate treated samples were related to vehicle control. n=3-6. Data are shown as mean ± SEM. For statistical analysis mixed-effect analysis (A) or one-way ANOVA (B) with Dunnett’s multiple comparisons test was used. *p<0.05 show significant difference between rocaglates and vehicle treatment.

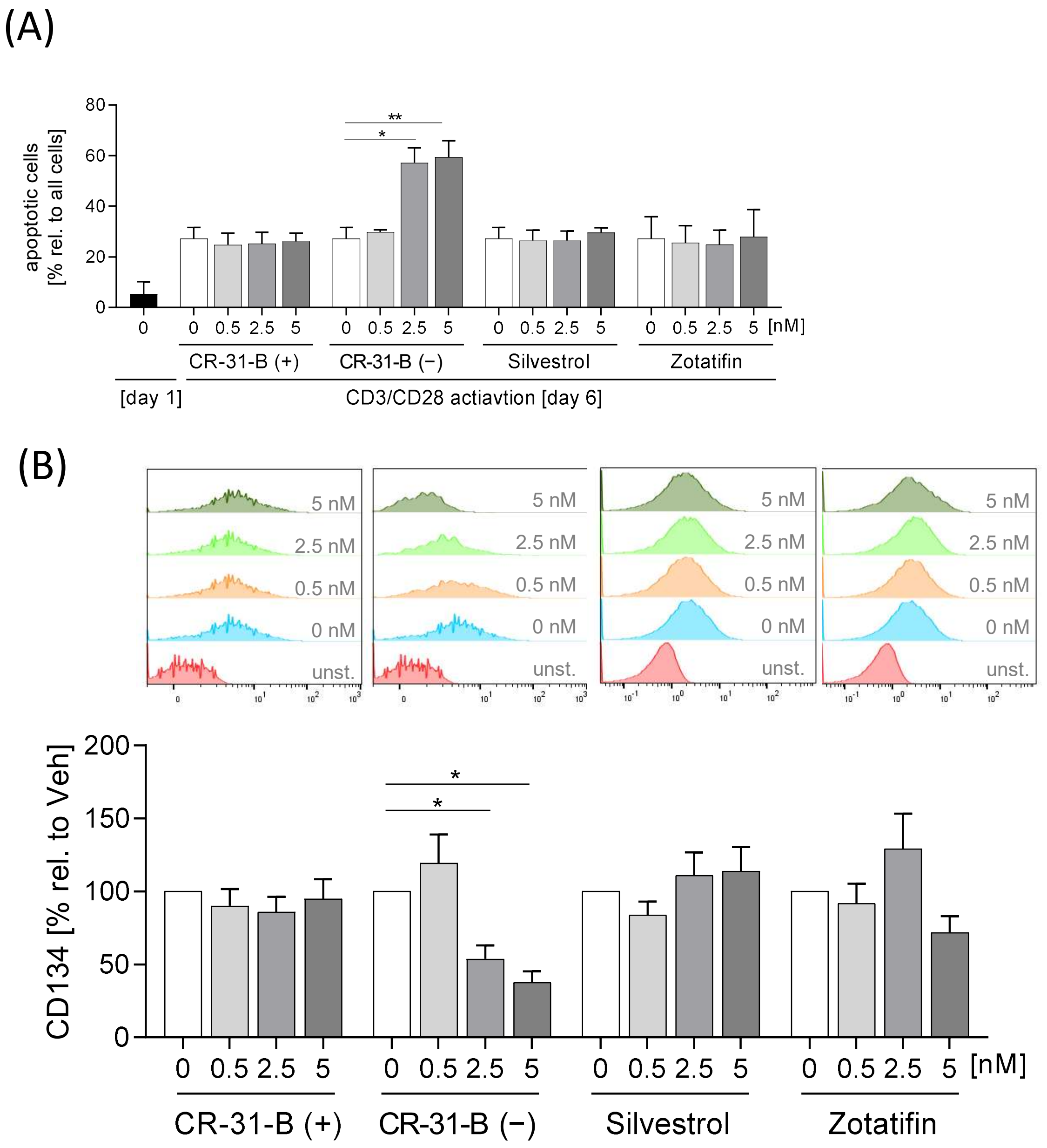

Supplemental Figure S13: Effect of rocaglates on T cell apoptosis (A) and on surface marker expression (B) of activated T cells. T cells isolated from buffy coats were activated with anti-CD3/anti-CD28 for 5 days. Apoptotic cells were identified with Annexin and 7-AAD staining. The apoptotic cells were related to all cells. The surface marker expression (MFI) was determined by flow cytometry. The geometric mean of the surface markers of rocaglate treated samples were related to the vehicle control. n=4. Data are shown as mean ± SEM. For statistical analysis two-way ANOVA with Dunnett's multiple comparisons test was used. *p<0.05, **p<0.01 show significant difference between rocaglates and vehicle treatment.

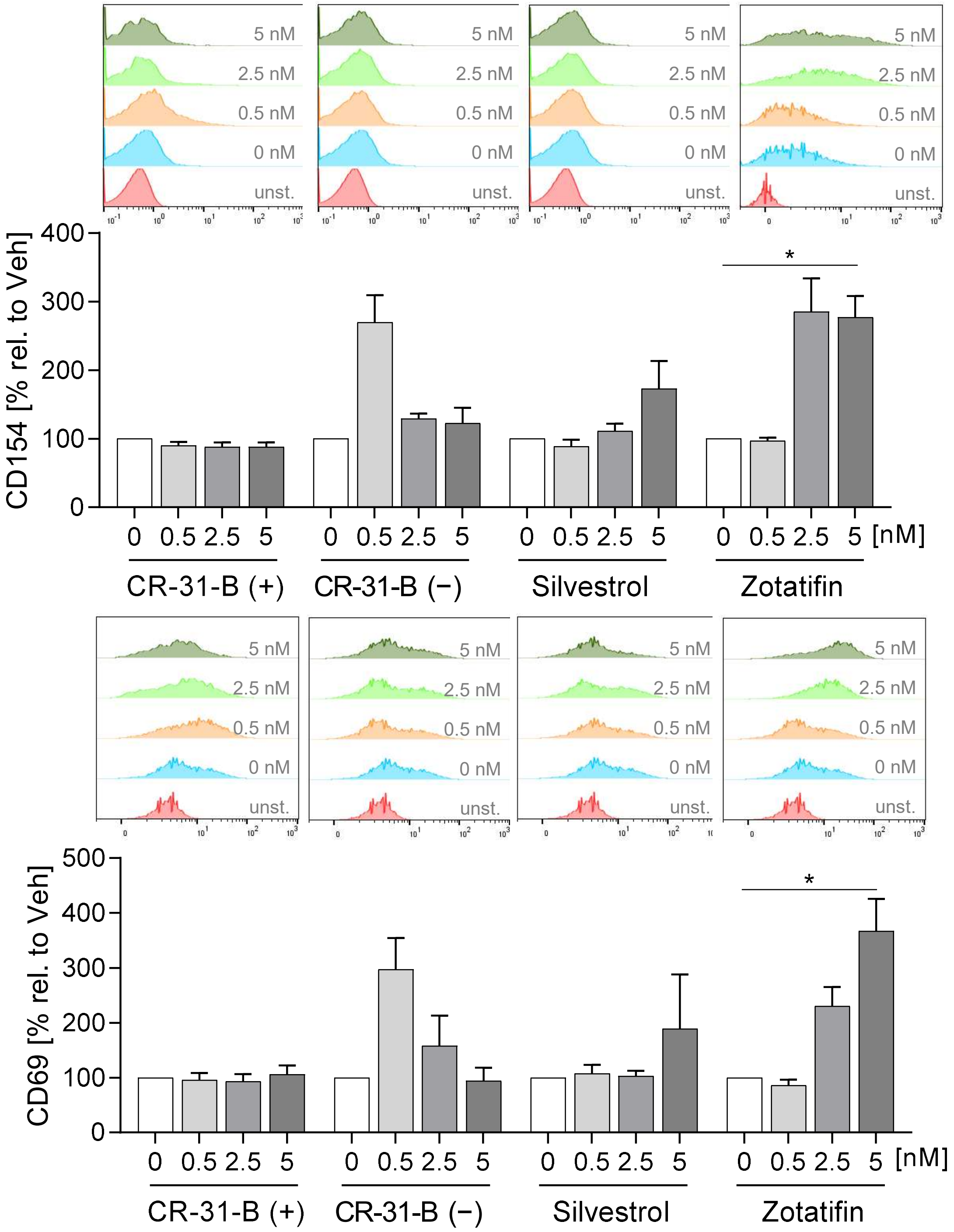

Continuation Supplemental Figure S13

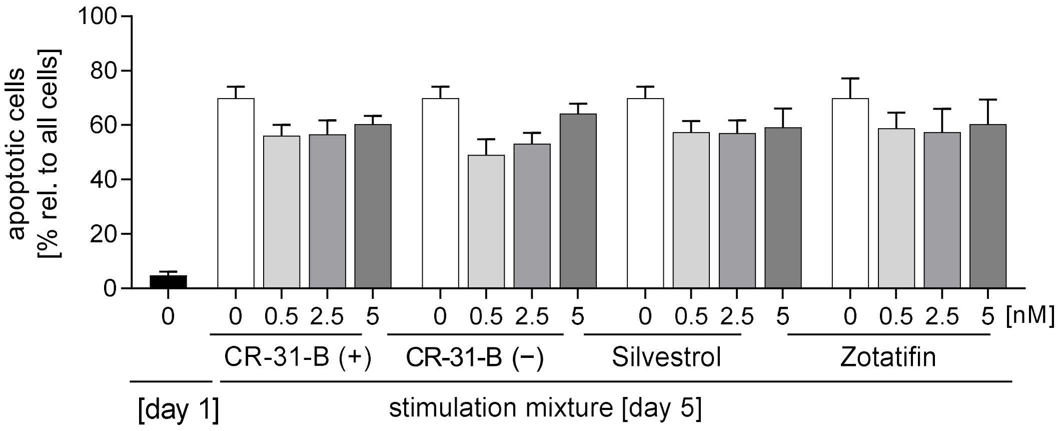

Supplemental Figure S14: Effect of rocaglates on B cell viability. B cells isolated from buffy coats were activated with 5 µl anti-IgM, 2.5 µg/ml CpG, 1 µg/ml sCD40L and 50 ng/ml IL12 for 5 days. Apoptotic cells were identified as cells positive for Annexin and 7-AAD staining. The apoptotic cells were related to all cells. n=3. Data are shown as mean ± SEM. For statistical analysis one-way ANOVA with Dunnett's multiple comparisons test was used. *p<0.05 show significant difference between rocaglates and vehicle treatment.

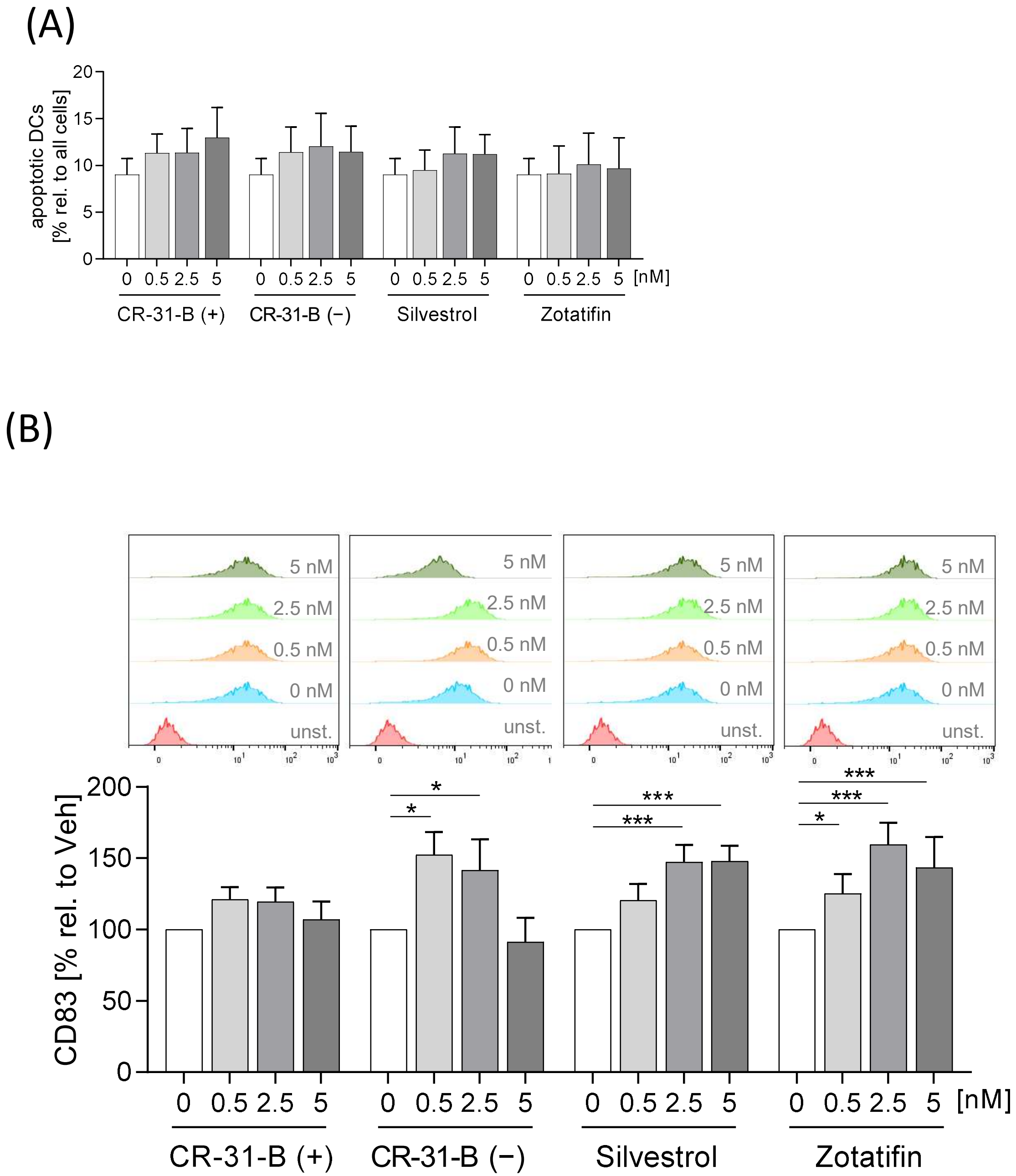

Supplemental Figure S15: Effect of rocaglates on apoptosis (A) and on surface marker expression (B) of activated DCs in the co-culture experiment. DCs isolated from buffy coats were activated with 10 ng/ml TNFα10 ng/ml IL6, 10 ng/ml IL1β and 1 µg/ml PGE_2 for 24h. Apoptotic cells were identified with Annexin and 7-AAD staining. The apoptotic cells were related to all cells. The surface marker expression (MFI) was determined by flow cytometry. The geometric mean of the surface markers of rocaglate treated samples were related to the vehicle control. n=4. Data are shown as mean ± SEM. For statistical analysis one-way ANOVA with Dunnett's multiple comparisons test was used. *p<0.05, **p<0.01, ***p<0.001 show significant difference between rocaglates and vehicle treatment.

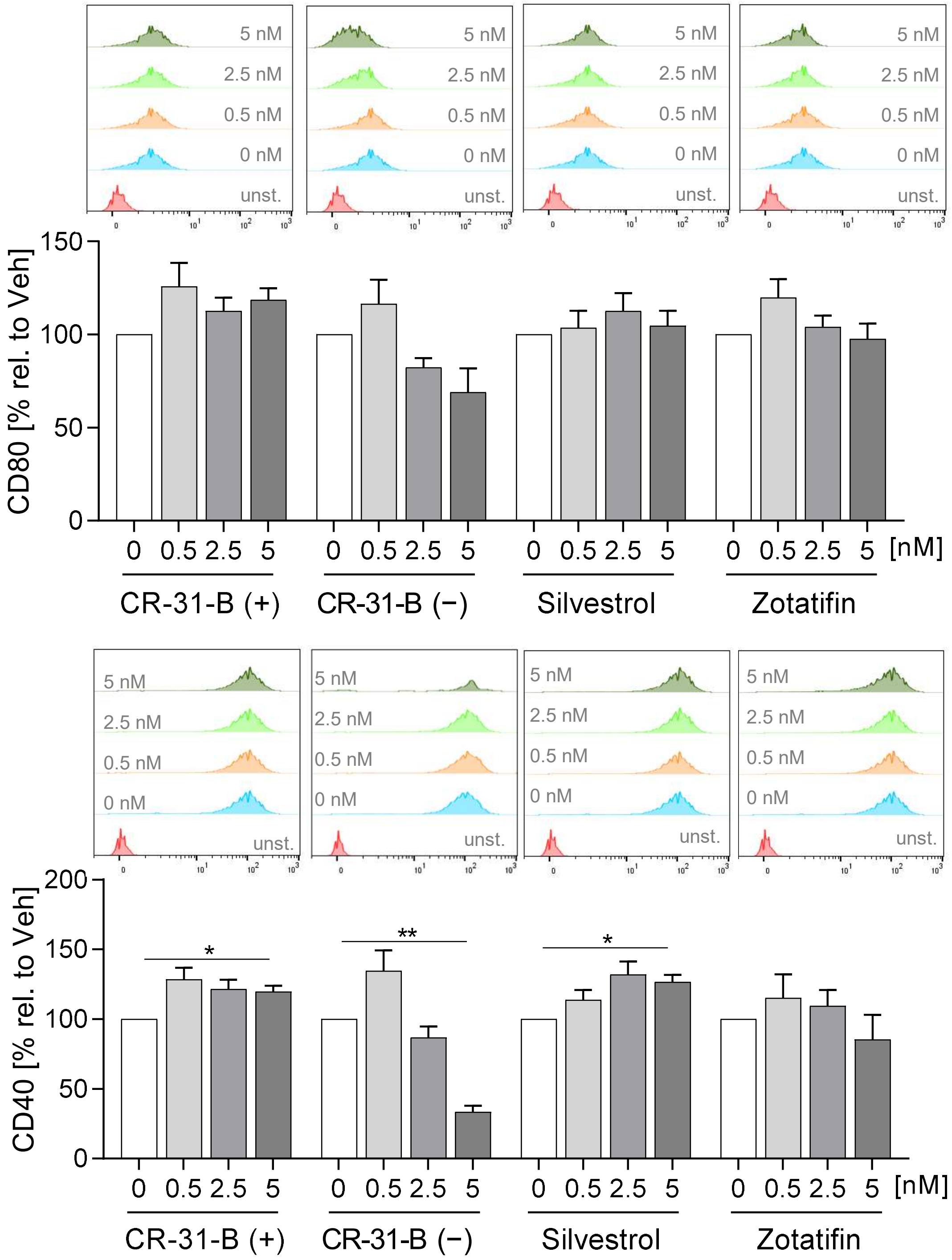

Continuation Supplemental Figure S15

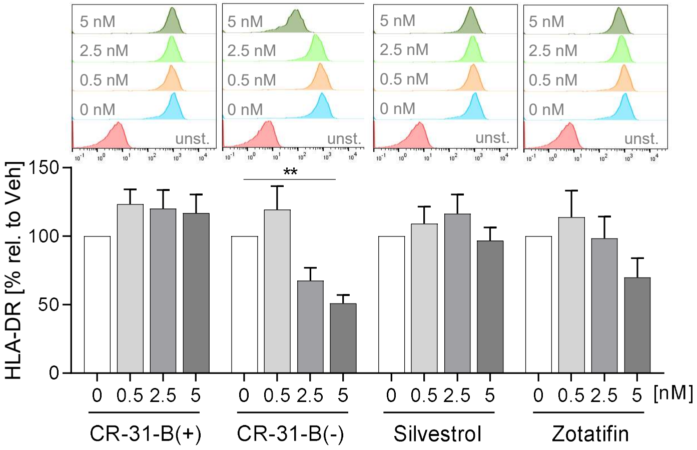

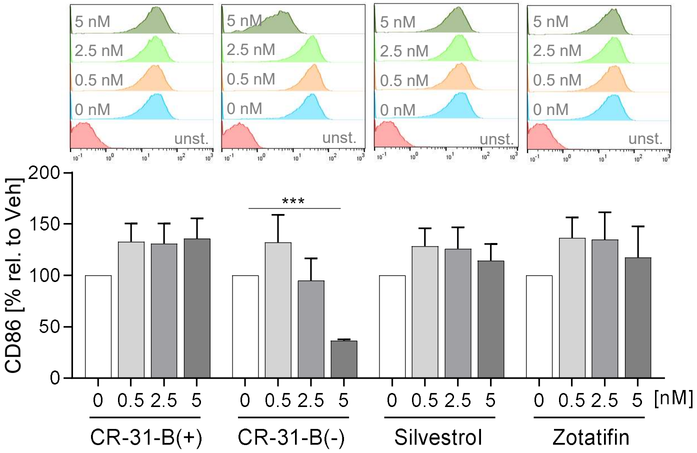

Continuation Supplemental Figure S15

(A)

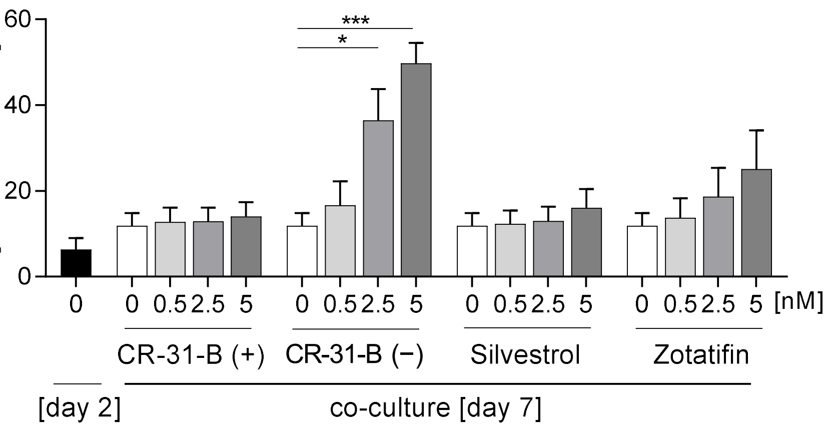

(B)

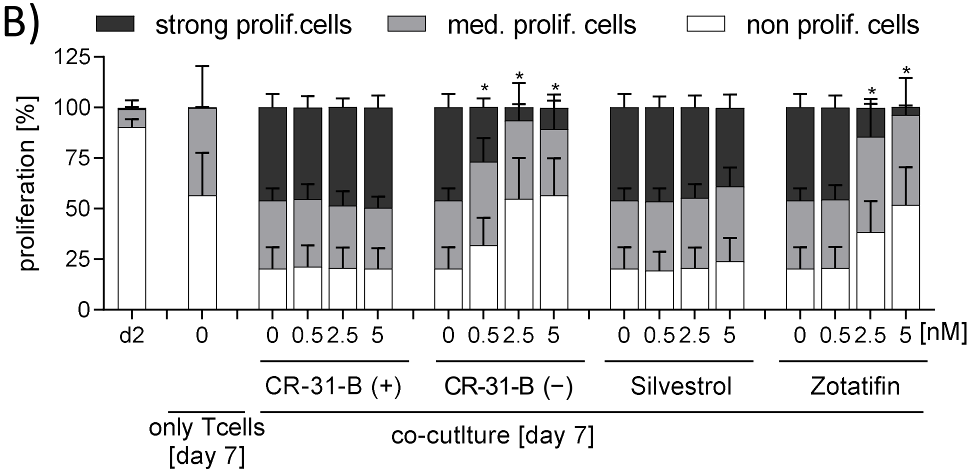

Supplemental Figure S16: Effect of rocaglates on T cell apoptosis (A), T cell proliferation (B) and on surface marker expression (C) of co-cultured T cells with

activated DCs. DCs isolated from buffy coats were activated with 10 ng/ml TNFα, 10 ng/ml IL6, 10 ng/ml IL1β and 1 µg/ml PGE_2 for 24h. The activated DCs were co-cultured with homologous T cells (labeled with CTV) for 5 days. Apoptotic cells were identified with Annexin and 7-AAD staining. The apoptotic cells were related to all cells. The proliferation was determined with the fluorescence lable CTV. The surface marker expression (MFI) was determined by flow cytometry. The geometric mean of the surface markers of rocaglate treated samples were related to the vehicle control. n=4. Data are shown as mean ± SEM. For statistical analysis one-way ANOVA with Dunnett’s multiple comparisons test was used. *p<0.05, ***p<0.001 show significant difference between rocaglates and vehicle treatment.

(C)

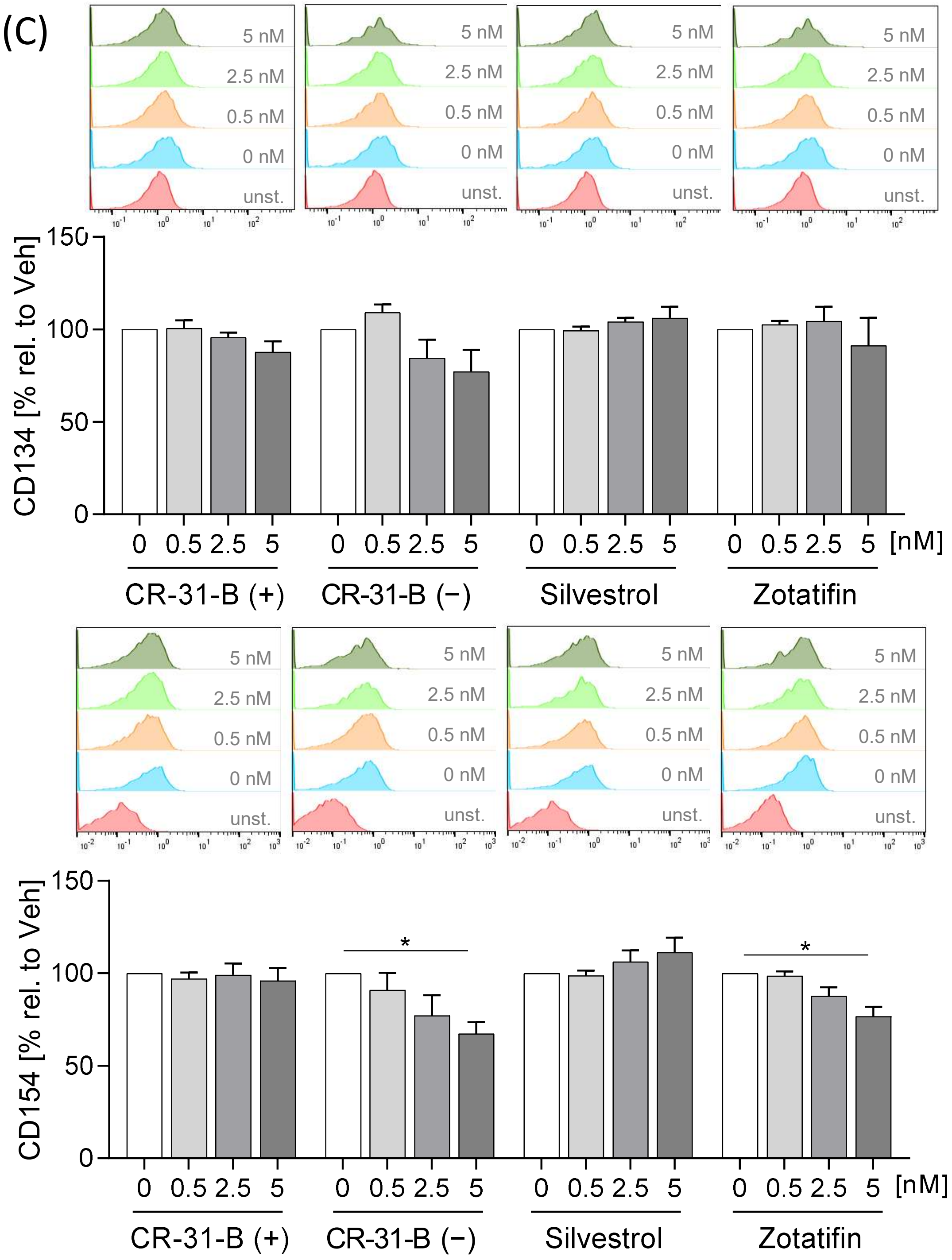

Continuation Supplemental Figure S16

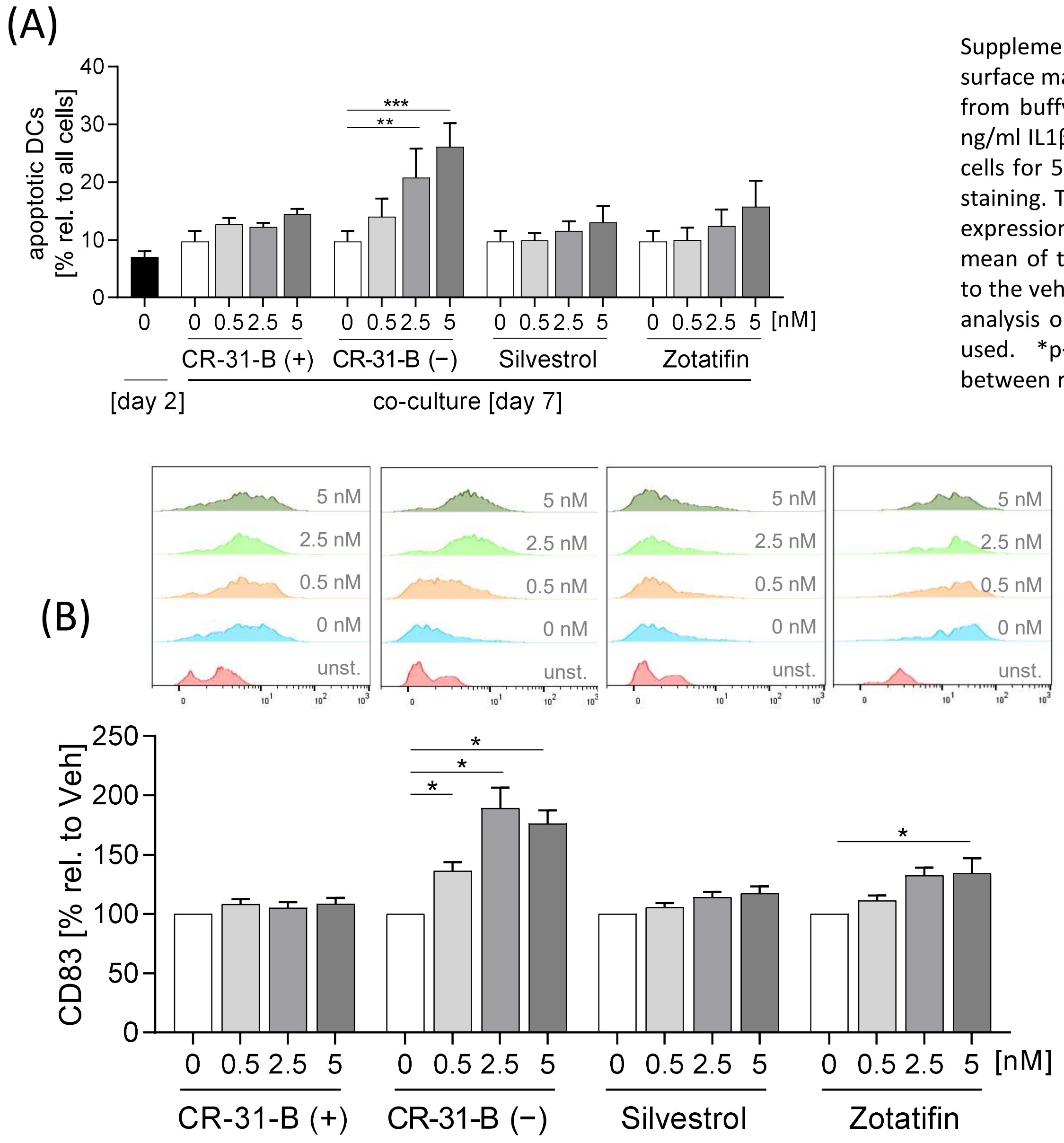

Supplemental Figure S17: Effect of rocaglates on apoptosis (A) and on surface marker expression (B) of co-cultured DCs with T cells. DCs isolated from buffy coats were activated with 10 ng/ml TNFα10 ng/ml IL6, 10 ng/ml IL1β and 1 µg/ml PGE_2 for 24h and co-cultured with homologous T cells for 5 days. Apoptotic cells were identified with Annexin and 7-AAD staining. The apoptotic cells were related to all cells. The surface marker expression (MFI) was determined by flow cytometry. The geometric mean of the surface markers of rocaglate treated samples were related to the vehicle control. n=4. Data are shown as mean ± SEM. For statistical analysis one-way ANOVA with Dunnett's multiple comparisons test was used. *p<0.05, **p<0.01, ***p<0.001 show significant difference between rocaglates and vehicle treatment.

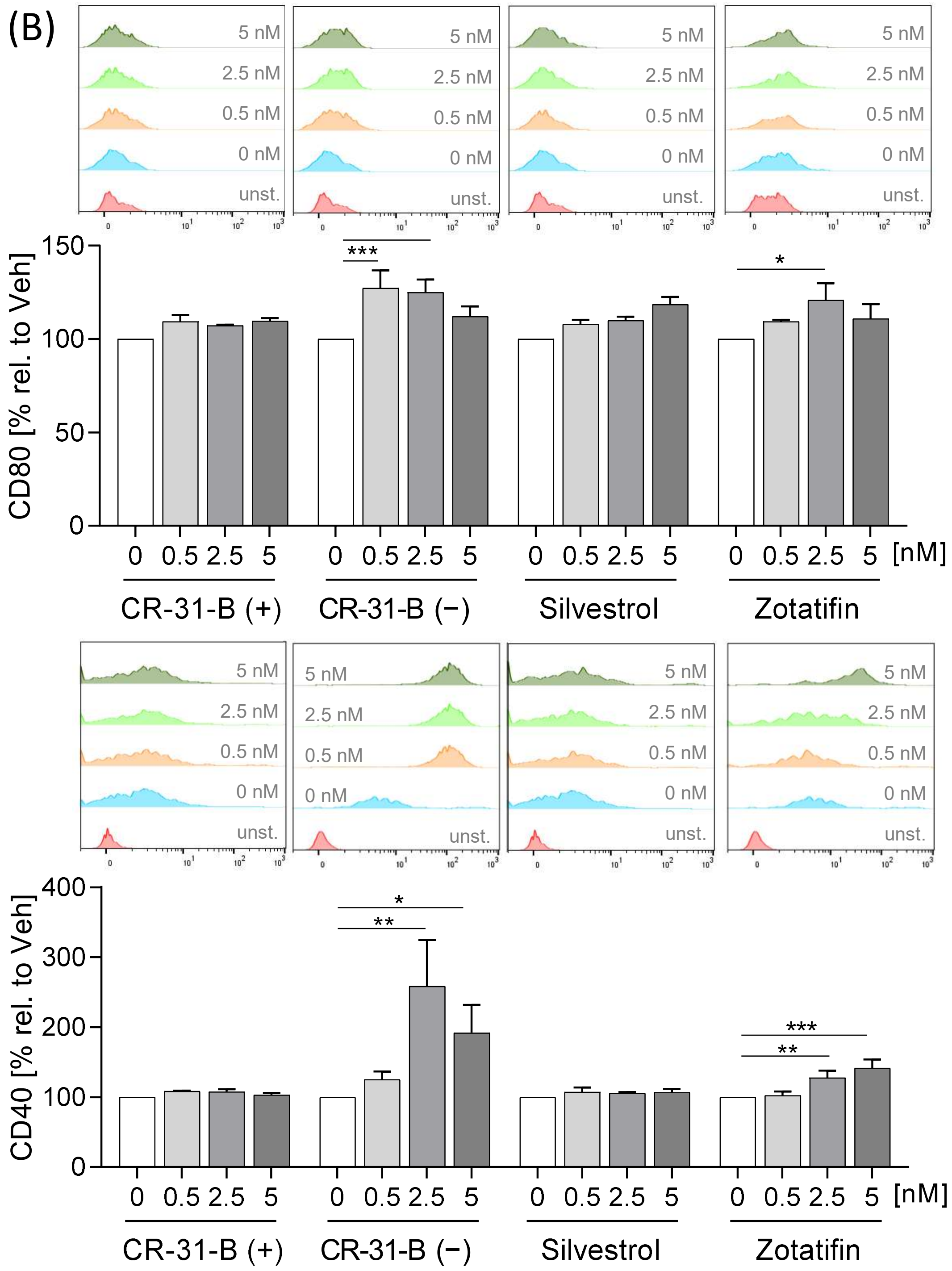

Continuation Supplemental Figure S17

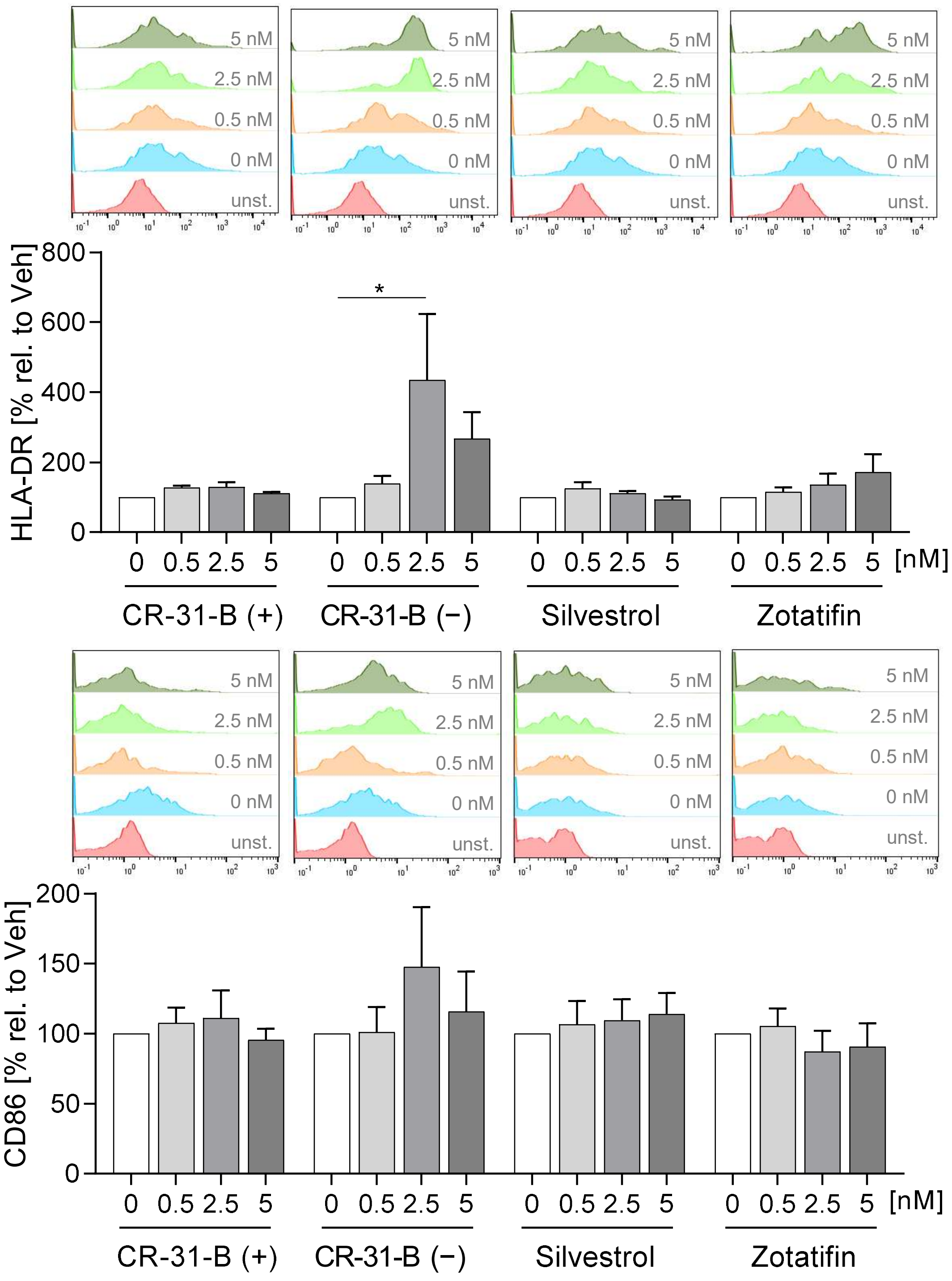

Continuation Supplemental Figure S17
